# TGF-β1-Licensed Murine MSCs Show Superior Therapeutic Efficacy in Modulating Corneal Allograft Immune Rejection *In Vivo*

**DOI:** 10.1016/j.ymthe.2020.05.023

**Published:** 2020-05-30

**Authors:** Kevin Lynch, Oliver Treacy, Xizhe Chen, Nick Murphy, Paul Lohan, Md Nahidul Islam, Ellen Donohoe, Matthew D. Griffin, Luke Watson, Steven McLoughlin, Grace O’Malley, Aideen E. Ryan, Thomas Ritter

**Affiliations:** 1 Discipline of Pharmacology and Therapeutics, School of Medicine, College of Medicine, Nursing and Health Sciences, National University of Ireland, Galway, Galway, Ireland; 2 Regenerative Medicine Institute (REMEDI), School of Medicine, College of Medicine, Nursing and Health Sciences, National University of Ireland Galway, Galway, Ireland; 3 Orbsen Therapeutics, National University of Ireland, Galway, Galway, Ireland; 4 CÚRAM, SFI Research Centre for Medical Devices, National University of Ireland Galway, Galway, Ireland

**Keywords:** mesenchymal stromal cells, cytokine licensing, TGF-β1, Tregs, immunomodulation, corneal transplantation, immunosuppression, autologous MSC therapy, PGE2, RNA sequencing

## Abstract

Mesenchymal stromal cells (MSCs) are a promising therapeutic option for multiple immune diseases/disorders; however, efficacy of MSC treatments can vary significantly. We present a novel licensing strategy to improve the immunosuppressive capacity of MSCs. Licensing murine MSCs with transforming growth factor-β1 (TGF-β MSCs) significantly improved their ability to modulate both the phenotype and secretome of inflammatory bone marrow-derived macrophages and significantly increased the numbers of regulatory T lymphocytes following co-culture assays. These TGF-β MSC-expanded regulatory T lymphocytes also expressed significantly higher levels of PD-L1 and CD73, indicating enhanced suppressive potential. Detailed analysis of T lymphocyte co-cultures revealed modulation of secreted factors, most notably elevated prostaglandin E2 (PGE2). Furthermore, TGF-β MSCs could significantly prolong rejection-free survival (69.2% acceptance rate compared to 21.4% for unlicensed MSC-treated recipients) in a murine corneal allograft model. Mechanistic studies revealed that (1) therapeutic efficacy of TGF-β MSCs is Smad2/3-dependent, (2) the enhanced immunosuppressive capacity of TGF-β MSCs is contact-dependent, and (3) enhanced secretion of PGE2 (via prostaglandin EP4 [E-type prostanoid 4] receptor) by TGF-β MSCs is the predominant mediator of Treg expansion and T cell activation and is associated with corneal allograft survival. Collectively, we provide compelling evidence for the use of TGF-β1 licensing as an unconventional strategy for enhancing MSC immunosuppressive capacity.

## Graphical Abstract

**Figure:**
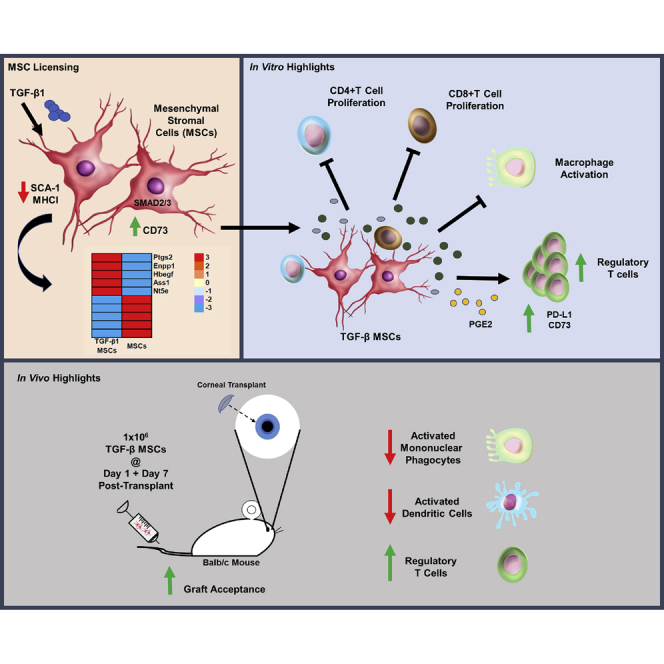

## Teaser

Ritter and colleagues evaluated the therapeutic efficacy of transforming growth factor (TGF)-β1-licensed mesenchymal stromal cells (MSCs) in an allogeneic murine corneal transplant model. TGF-β1 MSCs demonstrated potent immunosuppressive ability *in vitro* and significantly prolonged corneal allograft survival associated with prostaglandin E2 (PGE2)-mediated peripheral regulatory T cell expansion.

## Introduction

Full-thickness corneal transplantation (penetrating keratoplasty) is a last resort for patients suffering from dystrophic, degenerative, infectious, or inflammatory corneal disorders.^1^ During the last decade, however, improvements in ocular surgical concepts and techniques have led to the development of partial-thickness lamellar keratoplasty (for example, anterior and endothelial). Despite the increased rate of uptake of these newer procedures, penetrating keratoplasty remains the most frequent keratoplasty procedure performed worldwide, particularly for cases of deep-seated corneal infection and non-inflammatory-associated deep stromal scars, with approximately 185,000 full-thickness corneal transplant procedures performed worldwide in 2012 alone.^1^^,^^2^

While topical corticosteroids with or without adjuvant immunosuppressant therapy remains the gold standard treatment option for prolongation of allograft survival and, ultimately, tissue acceptance, patients are still highly susceptible to immune-mediated rejection.^3^ Limbal stem cell (LSC) infusion therapy is a possible treatment option in some cases; however, widespread use is restricted due to limited availability of sufficient numbers of LSCs and is not possible for patients with bilateral LSC deficiency.^4^ As a result, mesenchymal stromal cells (MSCs) have emerged as a viable therapeutic option due to the relative ease of isolating the cells and their high proliferation rates *in vitro*, generating high cell yields, as well as their immunomodulatory properties.^5^

MSCs are being extensively investigated in the context of immune modulation and suppression for the treatment of inflammatory disorders and for prolongation of allograft survival/tolerance induction in the setting of transplantation.^5^, ^6^, ^7^, ^8^, ^9^, ^10^ It is now well accepted that in order to fully harness MSC suppressive activity, the cells must be activated or licensed.^11^, ^12^, ^13^ This may occur *in vivo* due to inflammation or be pre-induced *in vitro* prior to downstream use.^11^, ^12^, ^13^ Multiple factors have been tested in an attempt to increase MSC efficacy in this regard. Examples include vitamin E,^14^ lipopolysaccharide (LPS),^15^ stromal-derived factor-1,^16^ migration inhibitory factor,^17^ and the pro-inflammatory cytokines tumor necrosis factor-α (TNF-α),^18^ interferon-γ (IFN-γ),^19^^,^^20^ interleukin-1β (IL-1β),^21^ either singly or in the combinations IL-1β + TNF-α^22^, ^23^, ^24^ and TNF-α + IFN-γ.^25^ Few studies, however, have investigated the effects of anti-inflammatory cytokine licensing on MSC therapeutic efficacy, particularly in the context of MSC immunosuppressive capacity.

Transforming growth factor-β1 (TGF-β1) is a pleiotropic molecule involved in various biological processes, including development, regulation of stem cell behavior, carcinogenesis, tissue homeostasis, and immune responses.^26^^,^^27^ Of the three known isoforms expressed by mammalian cells (TGF-β1, TGF-β2, TGF-β3), TGF-β1 is the isoform predominantly expressed by immune cells.^28^ Studies have shown that MSCs can secrete TGF-β1, which also plays a well-documented role in MSC immunomodulation, including a role in regulatory T cell (Treg) induction and/or expansion.^29^, ^30^, ^31^, ^32^ TGF-β1 is also known to directly influence MSCs, modulating their differentiation and migration.^33^^,^^34^ The vast majority of published studies investigating TGF-β pre-treatment in the context of MSCs either focus on the effects of TGF-β treatment on MSC differentiation capacity or on the use MSCs as a “carrier” for delivery of therapeutic levels of the cytokine to sites of injury via virus-mediated overexpression protocols.^35^, ^36^, ^37^, ^38^ These studies have identified a role for TGF-β1 in osteoblast differentiation and increased migratory capacity into remodeling sites following pre-activation with TGF-β, leading to synergistic bone formation and reabsorption.^36^ Lentivirus-mediated overexpression of TGF-β1 on MSCs showed improved renal function and reduced epithelial apoptosis and subsequent inflammation in a rat model of renal ischemia/reperfusion (I/R) injury via a mechanism not involving the direct canonical TGF-β1/Smad pathway.^37^ While such studies as highlighted above comprise the majority of published work related to TGF-β1-licensed MSCs, there are some reports investigating their immunomodulatory capacity. For example, de Witte et al.^39^ showed decreased susceptibility of TGF-β-licensed umbilical cord-derived MSCs to lysis by natural killer (NK) cells.

In the present study we sought to assess the effects of TGF-β1 conditioning on MSC immunomodulatory capacity in a mouse model of corneal transplantation. We show that MSCs pre-conditioned with TGF-β1 acquire unique potent immunosuppressive properties that distinguish them from pro-inflammatory cytokine pre-conditioned MSCs and confirm their therapeutic efficacy in a fully allogeneic model of murine corneal transplantation via the modulation of the allogeneic immune response. Selective antagonism of the prostaglandin E2 (PGE2) receptor EP4 (E-type prostanoid 4) revealed PGE2 signaling, through EP4, as the predominant mediator of TGF-β MSC-mediated immunosuppression.

## Results

### TGF-β Licensing Increases CD73 Expression on MSCs

**Figure 1:**
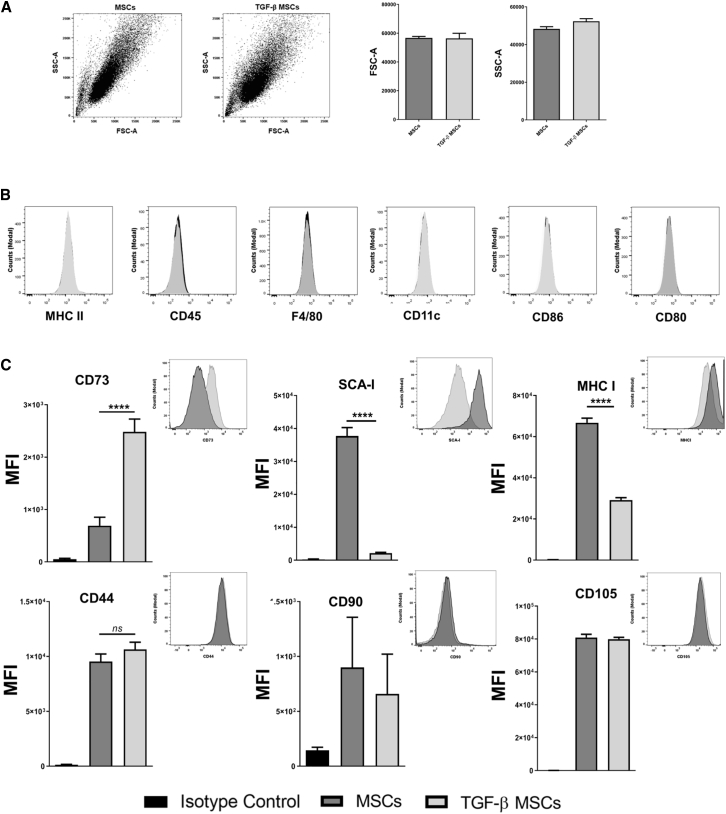
Pre-exposure to TGF-β Confers a Specific Phenotype on MSCs (A) Representative dot plots and bar graphs showing the FSC (cell size) and SSC (granularity) profile of MSCs versus TGF-β MSCs. (B) Representative flow cytometry histograms for the cell surface expression of negative MSC antigens MHC-II, CD45.2, F4-80, CD11c, CD80, and CD86. (C) Bar graphs showing median fluorescence intensity for the cell surface expression of positive MSC antigens CD105, CD73, SCA-1, CD90, CD44, and MHC-I (inset: representative histograms). Error bars show mean ± SD. ∗∗∗∗p < 0.0001, by one-way ANOVA, Tukey’s post hoc test (n = 3).

MSCs were isolated from femurs and tibias of female BALB/c mice and cultured as detailed in the Materials and Methods. Osteogenic (Figures S1A and S1B) and adipogenic (Figures S1C and S1D) differentiation capacities of the MSCs were confirmed prior to downstream use of the cells. TGF-β MSCs retained their progenitor phenotype as evidenced by their capacity to differentiate into both lineages (Figures S1A–S1D). The forward scatter/side scatter (FSC/SSC) profile of MSCs was assessed with and without TGF-β licensing to elucidate whether licensing had an effect on cell size (FSC) and/or granularity (SSC). As shown in Figure 1A, TGF-β licensing had no effect on either parameter. Flow cytometric assessment of lymphoid (CD45.2), myeloid (F4/80 and CD11c), transplant-associated (major histocompatibility complex class II [MHC-II]), and co-stimulatory markers revealed that TGF-β pre-conditioning had no significant effects on expression levels by MSCs (Figure 1B). Furthermore, we observed no significant changes in the expression levels of the MSC characterization markers CD44, CD90, and CD105 (Figure 1C). High levels of MHC class I (MHC-I) expression were seen on untreated MSCs but MHC-I expression was significantly lower on their TGF-β-licensed counterparts (Figure 1C). This finding is in contrast to the reported effects of licensing strategies using pro-inflammatory cytokines on MHC-I expression.^40^ We also noted that TGF-β-licensed MSCs expressed significantly lower levels of the identification marker stem cell antigen (SCA)-1. Finally, we observed a significant increase in CD73 expression following TGF-β licensing, which has particular relevance to the current study due to the reported role played by CD73 in immune tolerance.^41^, ^42^, ^43^ These data illustrate that TGF-β MSCs have a particular phenotype that is distinguishable from unlicensed MSCs, and we next sought to investigate how these cells would influence T lymphocytes in syngeneic co-culture assays.

### TGF-β Licensed MSCs Suppress Syngeneic T Cell Proliferation and Significantly Expand Total Treg Numbers

**Figure 2:**
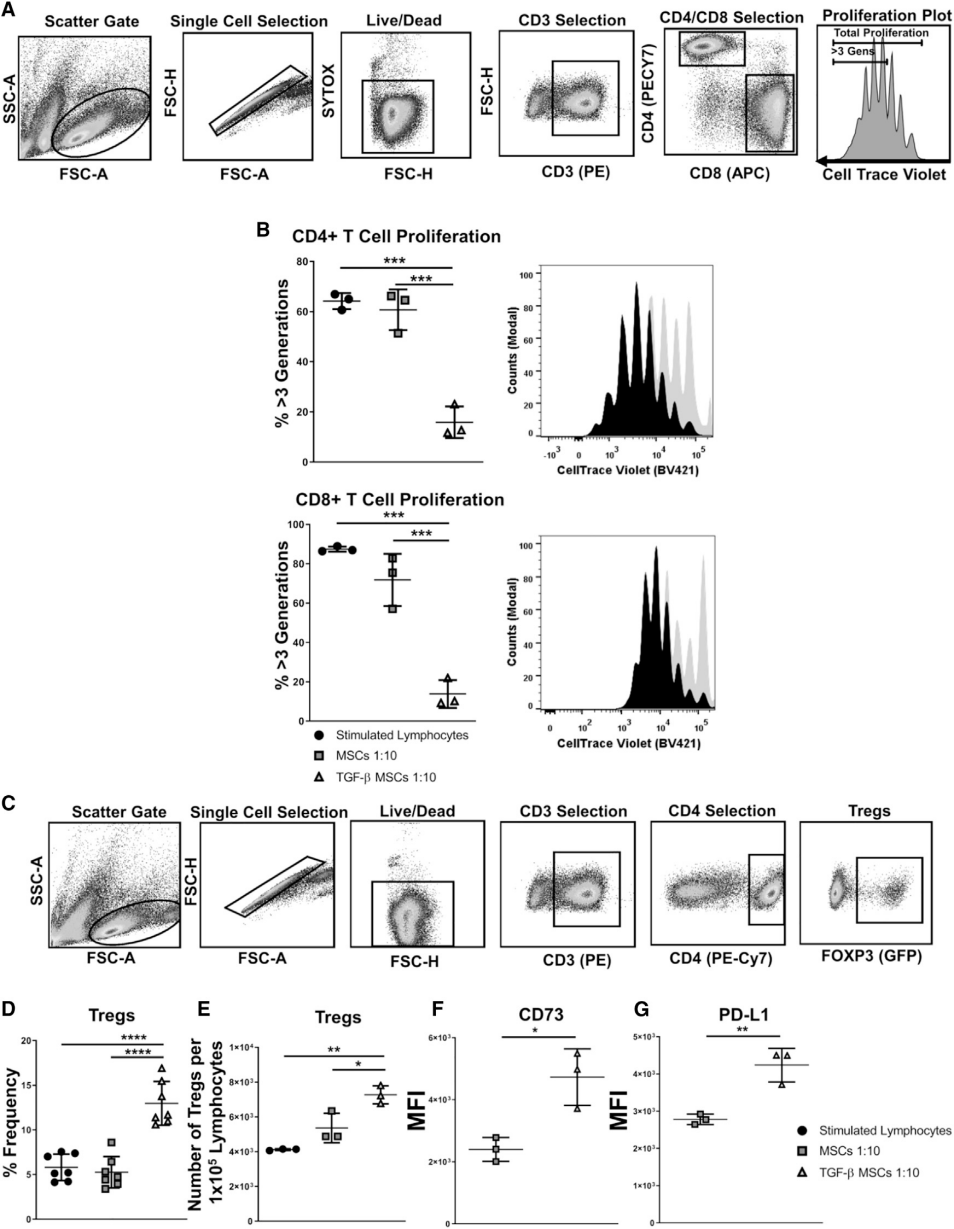
TGF-β MSCs Inhibit T Cell Proliferation while Inducing/Expanding Regulatory T Cells in Syngeneic *In Vitro* Co-culture Assays Untreated or TGF-β MSCs were co-cultured with T lymphocytes at a ratio of 1:10 (MSCs/lymphocytes) for 96 h in the presence of anti-CD3/CD28 polyclonal-stimulating beads. CTV was used to determine lymphocyte proliferation. (A) Flow cytometry gating strategy used to assess CD4^+^ or CD8^+^ T cell proliferation. (B) Bar charts and histograms showing CD4^+^ and CD8^+^ T cell proliferation for more than three generations and representative flow cytometry plot overlays. (C) Flow cytometry gating strategy used to select CD4^+^Foxp3^+^ Tregs. (D) Frequency (%) of CD4^+^Foxp3^+^ Tregs after co-culture with untreated or TGF-β MSCs. (E) Total number of Tregs per 1 × 10^5^ lymphocytes after co-culture with untreated or TGF-β MSCs. (F and G) Median fluorescence intensity (MFI) of CD73 (F) and PD-L1 expression on Tregs (G) following 96 h of co-culture. Error bars show mean ± SD. ∗p < 0.05, ∗∗p < 0.01, ∗∗∗p < 0.001, ∗∗∗∗p < 0.0001, by multiple unpaired, two-tailed Student’s t test and one-way ANOVA, Tukey’s *post hoc* test (n = 3–7).

To assess the immunomodulatory effects of TGF-β, MSCs with or without TGF-β pre-conditioning were co-cultured with anti-CD3/CD28-activated lymphocytes derived from lymph nodes and spleens of BALB/c mice. The flow cytometric gating strategy followed by further discrimination of live, non-aggregated cells and specific lymphocyte identification by CD3, CD4, and CD8 is shown in Figure 2A. To assess the effect of licensed MSCs on T cell proliferation, TGF-β MSCs were placed in co-culture assays with anti-CD3/CD28-stimulated lymphocytes. Proliferation of CD4^+^ and CD8^+^ lymphocytes was analyzed by flow cytometry using CellTrace Violet (CTV) dye. The results demonstrated that TGF-β MSCs markedly reduced the proliferation (the data represent % proliferation for more than 3 generations) of both CD4^+^ (15.85% ± 6.3% SD) (Figure 2B, upper left and right) and CD8^+^ (13.83% ± 7.09% SD) (Figure 2B, lower left and right) T lymphocytes compared to unlicensed MSCs (60.73% ± 8.12% SD for CD4^+^ and 71.8% ± 13.24% SD for CD8^+^) (Figure 2B, upper and lower). We next sought to investigate the effects of TGF-β MSCs on Tregs. Utilizing the Foxp3-EGFP transgenic BALB/c mouse, Treg proportions were assessed by flow cytometry (see Figure 2C for gating strategy). We found that TGF-β MSCs significantly increased the frequency of Tregs following lymphocyte co-culture when compared to unlicensed MSCs (Figure 2D). Furthermore, we assessed whether the increase in Treg frequency was due to an increase in the absolute number of Tregs or whether it was due to an increase in the ratio between Tregs and conventional T cells. The data showed that TGF-β MSCs significantly increased the absolute numbers of Tregs (as calculated per 1 × 10^5^ live lymphocytes) (Figure 2E). Due to their previously documented role in mediating Treg immunosuppression,^44^^,^^45^ we next assessed expression of CD73 and PD-L1 on Tregs. CD73 expression was significantly increased following direct contact of lymphocyte co-culture with TGF-β MSCs compared to untreated MSCs (median fluorescence intensity [MFI], 4,733 ± 914 SD for TGF-β MSCs versus 2,399 ± 385 SD for untreated MSCs) (Figure 2F). We also observed a significant increase in PD-L1 expression on Tregs following co-culture with TGF-β MSCs (MFI, 4,240 ± 451 SD for TGF-β MSCs versus 2,783 ± 139 SD for untreated MSCs) (Figure 2G). These findings suggest that TGF-β MSCs induce expansion of Tregs with an enhanced immunomodulatory potential. To eliminate the possibility that the expansion of Tregs induced by TGF-β MSCs is due to TGF-β sequestering, we assessed the expression of latency-associated peptide (LAP) and glycoprotein A repetitions predominant (GARP) on MSCs, as we had shown that TGF-β MSCs did not secrete TGF-β (Figure S2A). It has been shown that GARP binds LAP/TGF-β1 to the cell surface of activated Tregs.^46^ We therefore assessed expression of LAP/GARP, both of which regulate TGF-β function. We observed no significant difference in expression levels between untreated and TGF-β MSCs, concluding that TGF-β MSC-mediated immunosuppression and Treg induction were independent of MSC-derived TGF-β (Figure S2B).

### TGF-β MSCs Induce Immunosuppression *In Vitro* Independent of Nitric Oxide (NO) Production

**Figure 3:**
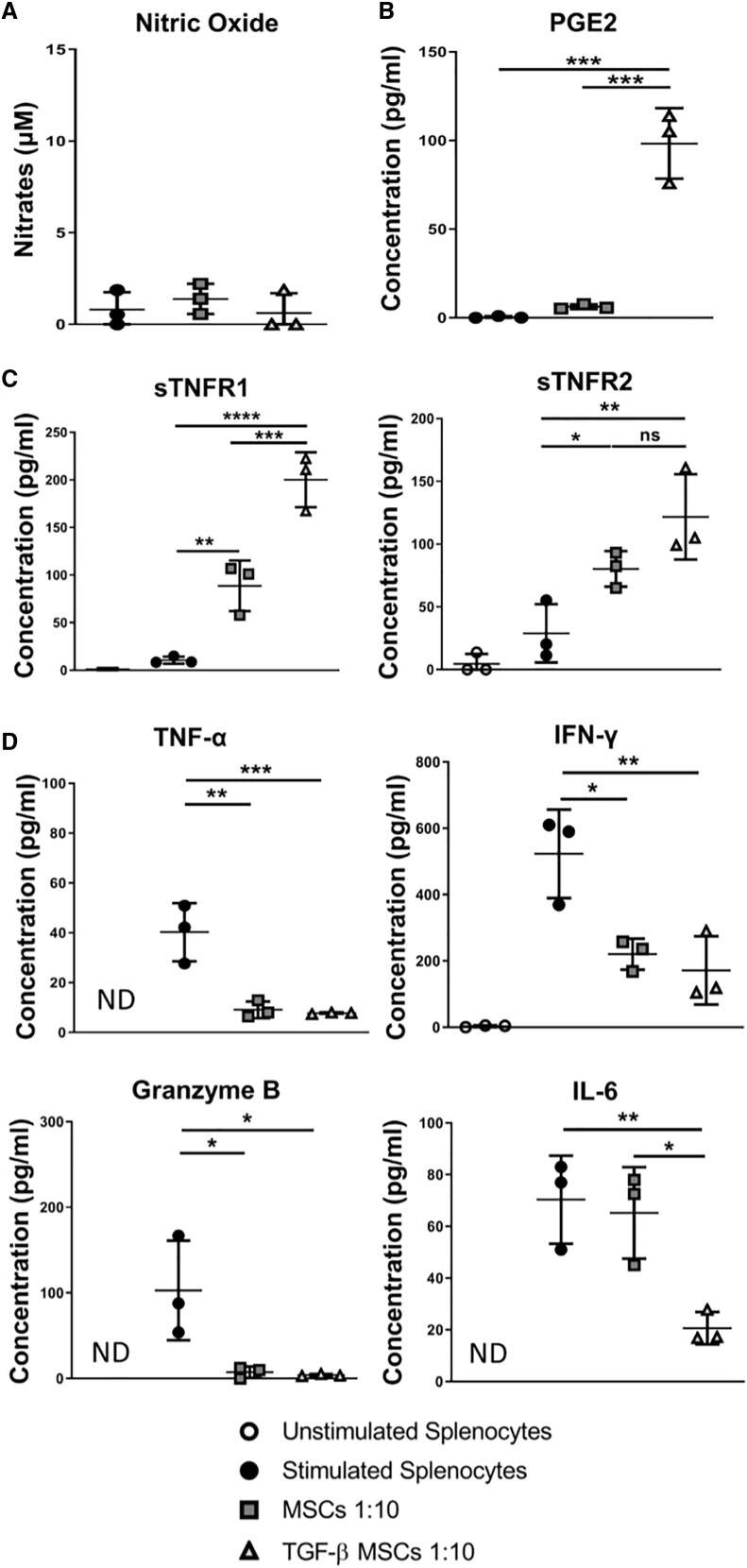
TGF-β MSCs Establish an Immunosuppressive Microenvironment Unrelated to Nitric Oxide (NO) Production Untreated or TGF-β MSCs were co-cultured with T lymphocytes at a ratio of 1:10 (MSCs/lymphocytes) for 96 h in the presence of anti-CD3/CD28 polyclonal-stimulating beads. (A–D) Supernatants were collected and centrifuged at 1,000 × *g* for 5 min to remove cell debris and then analyzed by (A) a Griess assay to measure nitrates, (B) an ELISA to measure PGE2, and (C and D) a magnetic Luminex assay to measure (C) sTNFR1 and sTNFR2 and (D) TNF-α, IFN-γ, granzyme B, and IL-6. Supernatants were used neat. Error bars show mean ± SD. ∗p < 0.05, ∗∗p < 0.01, ∗∗∗p < 0.001, ∗∗∗∗p < 0.0001, by one-way ANOVA, Tukey’s *post hoc* test (n = 3).

Numerous published studies have attributed the immunosuppressive capacity of rodent MSCs to increases in NO production.^13^^,^^47^^,^^48^ However, using the alternative licensing strategy reported herein using TGF-β, we did not detect an increase in nitrate concentration in co-culture supernatants above baseline levels (Figure 3A). Instead, we observed a significant increase in PGE2 secretion in TGF-β MSCs compared to unlicensed MSCs (Figure 3B). While PGE2-mediated immunomodulation by MSCs has been reported in the published literature,^47^^,^^49^, ^50^, ^51^, ^52^ to the best of our knowledge, this is the first time such an increase has been reported in response to TGF-β pre-conditioning coupled with no detectable increase in NO production. Due to the well-established role played by TNF receptor 2 (TNFR2)^53^, ^54^, ^55^ and purported role played by TNFR1^56^ in the activation and expansion of Tregs, we analyzed the concentrations of the soluble forms of these two receptors following co-culture with syngeneic lymphocytes. The results showed significantly increased levels of soluble (s)TNFR1 and a slight increase in sTNFR2 in wells containing TGF-β MSCs, indicating higher levels of shedding of sTNFRs by cells in co-cultures when TGF-β MSCs are present (Figure 3C).

Importantly, however, note that levels of TNF-α remained unchanged following co-culture with MSCs (licensed or not) (Figure 3D). We also analyzed the additional key pro-inflammatory cytokines IFN-γ and IL-6 and the apoptosis-inducing serine protease granzyme B, secreted by cytotoxic T lymphocytes and natural killer cells primarily, and found in all cases significantly less secretion following co-culture with TGF-β MSCs (Figure 3D) compared to stimulated lymphocytes alone. IL-6 levels were significantly reduced in TGF-β MSC co-cultures compared to unlicensed MSCs (Figure 3D). Indeed, Lin et al.^57^ showed that addition of IL-6 to cell culture medium could decrease Treg frequency. These findings suggest that reduced IL-6 may be associated with the increased frequency of Tregs observed following co-culture with TGF-β MSCs (Figure 2D).

### The Transcriptional Profile of TGF-β MSCs Is Distinct from MSCs, and It Identifies PGE2 and CD73 as Predominant Mediators in TGF-β MSC Immunomodulation

**Figure 4:**
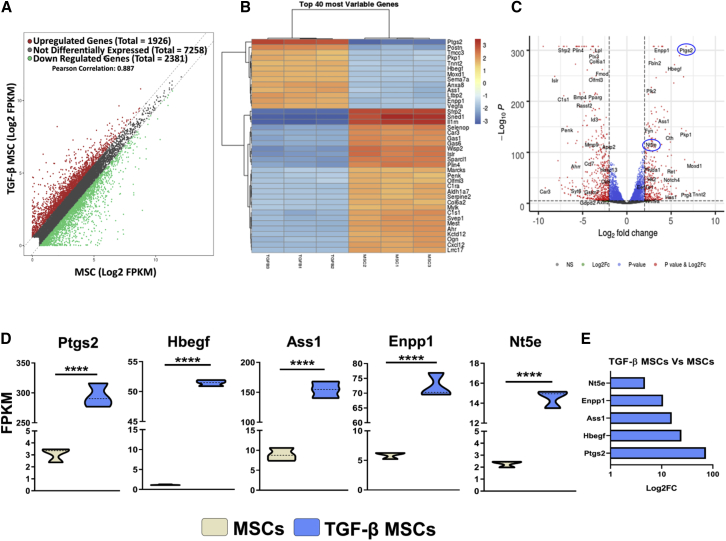
Gene Expression Profile of TGF-β MSCs Compared to Untreated MSCs MSCs were pre-treated with TGF-β for 72 h or untreated as detailed previously. RNA was isolated and RNA sequencing was performed as detailed in the Materials and Methods. (A) Scatterplot of differentially expressed genes. The scatterplot was generated by comparing genes expressed by MSCs with TGF-β MSCs with log_2_-scaled fragments per kilobase of transcript per million mapped reads (FPKM) values. (B) Heatmap highlighting the 40 most differentially expressed genes between MSCs (n = 3) and TGF-β MSCs (n = 3). The changing intensity of color refers to how upregulated (red) or downregulated (blue) the various genes are. (C) Volcano plot of upregulated or downregulated genes between MSCs and TGF-β MSCs, with genes involved in PGE2 synthesis and CD73 expression highlighted. (D) Violin plots showing differential FPKM for *Ptgs2*, *Hbegf*, *Ass1*, *Enpp1*, and *Nt5e*. Changes in FPKM values were observed for *Ptgs2*, *Hbegf*, *Ass1*, *Enpp1*, and *Nt5e* between MSCs and TGF-β MSCs. Dashed lines indicate median values. ∗∗∗∗p< 0.0001, by unpaired two-tailed t test. (E) Log_2_ fold changes for *Ptgs2*, *Hbegf*, *Ass1*, *Enpp1*, and *Nt5e* between MSCs and TGF-β MSCs.

Following the observation of the enhanced immunosuppressive ability of TGF-β MSCs *in vitro*, we next sought to investigate the differences in the transcriptional profiles between MSCs and TGF-β MSCs in order to elucidate the molecular mechanism of action. From RNA sequencing (RNA-seq) of TGF-β and unlicensed MSCs, we observed that a total of 1,926 genes were upregulated, 2,381 genes were downregulated, and 7,258 genes were non-differentially expressed (Figure 4A), highlighting a distinct transcriptional profile between MSCs and TGF-β MSCs. A heatmap of the 40 most variable genes (Figure 4B) along with a volcano plot of upregulated or downregulated genes (Figure 4C) between MSCs and TGF-β MSCs highlighted changes in many genes related to immune regulation, tissue repair, cell proliferation, cytoprotection, or migration. Of particular interest in the current study was the finding that prostaglandin-endoperoxide synthase 2 (*Ptgs2*), heparin binding EGF-like growth factor (*Hbegf*), argininosuccinate synthetase 1 (*Ass1*), ectonucleotide pyrophosphatase/phosphodiesterase 1 (*Enpp1*), and 5′nucleotidase, ecto (*Nt5e*, CD73) were all significantly upregulated in TGF-β MSCs. *Ptgs2*, also known as cyclooxygenase-2, was the most differentially expressed gene (73-fold increase) by TGF-β MSCs (Figures 4D and 4E). Cyclooxygenase-2 is a key enzyme in prostaglandin biosynthesis^58^ and aligns with our observed increase of PGE2 in TGF-β MSC/T lymphocyte co-culture experiments (Figure 3B). *Hbegf* is a protein coding gene whose gene product is a mitogen for fibroblasts^59^ and stimulates proliferation and migration^60^ of MSCs. It also has cytoprotective attributes protecting MSCs from apoptosis^60^ and was increased 24-fold in TGF-β MSCs (Figures 4D and 4E). *Ass1* encodes an enzyme that catalyzes the second last step of the arginine biosynthesis pathway. In myeloid cells, arginine is metabolized either by NO synthases or by arginases, and the fate of arginine metabolism is an integral regulator of innate and adaptive immune responses,^61^^,^^62^ where NO metabolism is linked to a pro-inflammatory outcome and arginine is linked to an anti-inflammatory outcome. *Ass1* was 15-fold upregulated in TGF-β MSCs (Figures 4D and 4E). *Enpp1* encodes an ecto-nucleotide that converts extracellular ATP to adenosine monophosphate (AMP).^63^ This is a process in adenosine synthesis and has been described as one of the many mechanisms by which MSCs modulate immune cells. A 10-fold increase in *Enpp1* was observed in TGF-β MSCs (Figures 4D and 4E). *Nt5e* was 4.7-fold upregulated in TGF-β MSCs (Figures 4D and 4E). The gene product of *Nt5e* is CD73 and this molecule converts extracellular nucleotides such as AMP into membrane-permeable nucleosides such as ADO.^64^ This fold increase in the gene for CD73 confirmed our previously observed increased expression of cell surface CD73 (Figure 1C). In contrast to MSCs, genes involved in cell cycle, DNA replication, ribosome biogenesis, cytoprotection, and mRNA processing were among the most highly upregulated genes in TGF-β MSCs (Figure S3A). We also observed that upregulation of *Ptgs2*, *Hbegf*, and *Enpp1* were partially localized to the cell membrane with *Ptgs2* and *Ass1* co-localized to the nucleus and cytoplasm (Figure S3B). The main findings from this analysis suggest that CD73 and PGE2 are the predominant mediators of TGF-β MSC immunomodulation. The data discussed herein have been deposited in NCBI’s Gene Expression Omnibus^65^ and are accessible through GEO series accession no. GSE150008 (https://www.ncbi.nlm.nih.gov/geo/query/acc.cgi?acc=GSE150008).

### Investigation of Mechanisms of Action, Using Transwell and Pharmacological Inhibition Approaches, Suggests That TGF-β MSC Immunosuppression Is Mediated by Cell Contact-Dependent Mechanisms and Requires Signaling through the EP4 Receptor

**Figure 5:**
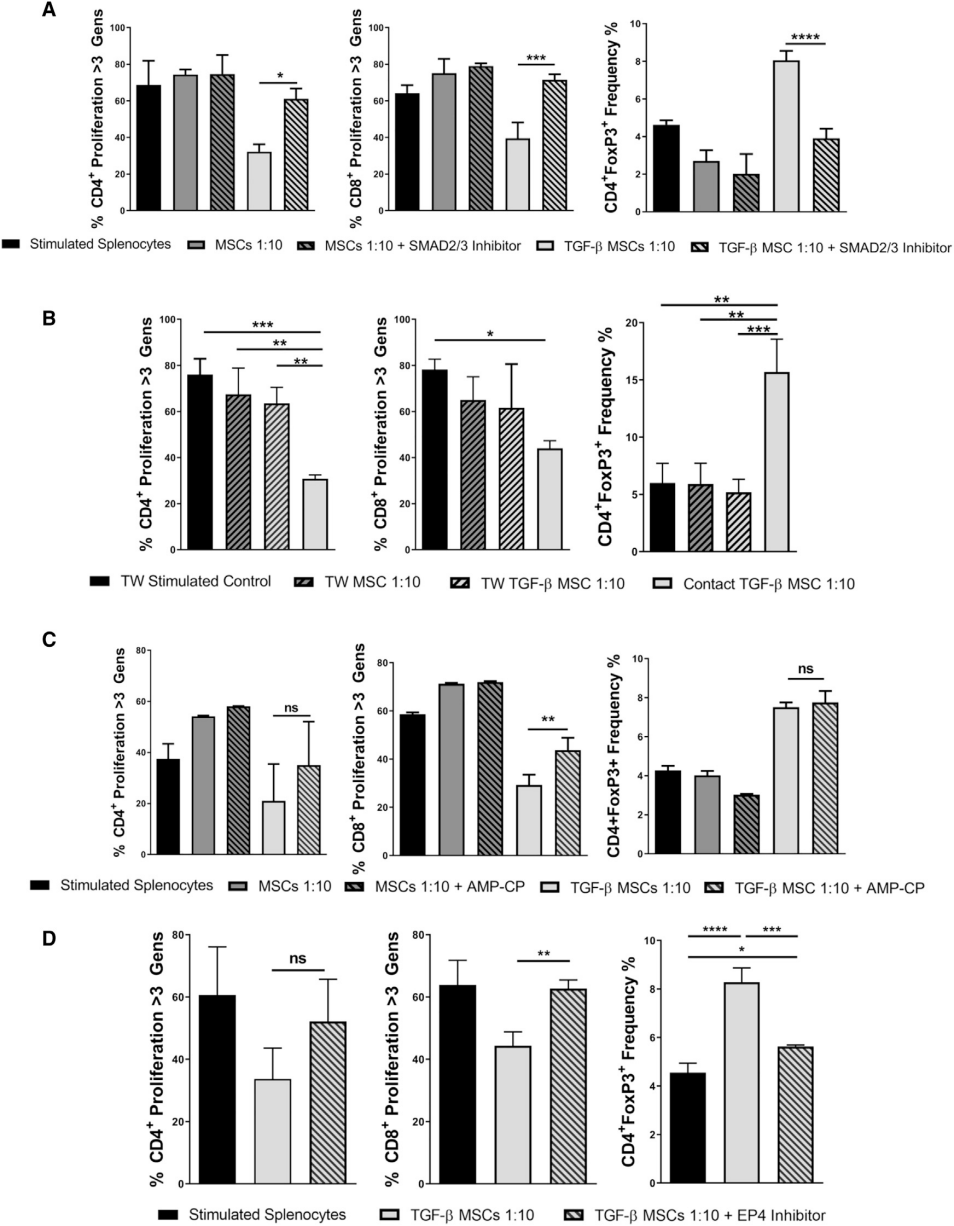
Potent TGF-β MSC Immunosuppressive Ability Is Dependent on Cell-to-Cell Contact-Mediated Smad2/3 Signaling Requiring Successful PGE2/EP4 Binding (A) Untreated or TGF-β MSCs with or without Smad2/3 inhibitor were co-cultured with T lymphocytes at a ratio of 1:10 (MSCs/lymphocytes) for 96 h in the presence of anti-CD3/CD28 polyclonal-stimulating beads. CTV was used to determine lymphocyte proliferation. Bar charts show CD4^+^ and CD8^+^ T lymphocyte proliferation for more than three generations and frequency (%) of CD4^+^Foxp3^+^ cells in co-culture wells. (B) Untreated or TGF-β MSCs were seeded in the top compartment of a 0.8-μm transwell, and polyclonally stimulated lymphocytes were placed in the bottom compartment in a 24-well flat-bottom plate and co-cultured for 96 h. Bar charts show CD4^+^ and CD8^+^ T lymphocyte proliferation for more than three generations and frequency (%) of CD4^+^Foxp3^+^ cells in co-culture wells. (C) Untreated or TGF-β MSCs with or without AMP-CP were co-cultured with polyclonally stimulated T lymphocytes for 96 h. Bar charts show CD4^+^ and CD8^+^ T lymphocyte proliferation for more than three generations and frequency (%) of CD4^+^Foxp3^+^ cells in co-culture wells. (D) TGF-β MSCs with or without EP4 receptor antagonist were co-cultured with polyclonally stimulated T lymphocytes for 96 h. Bar charts show CD4^+^ and CD8^+^ T lymphocyte proliferation for more than three generations and frequency (%) of CD4^+^Foxp3^+^ cells in co-culture wells. Error bars show mean ± SD. ∗p < 0.05, ∗∗p < 0.01, ∗∗∗p < 0.001, ∗∗∗∗p < 0.0001, by one-way ANOVA, Tukey’s *post hoc* test (n = 3).

To investigate the requirement of Smad2/3 in mediating TGF-β licensing, we performed an MSC/splenocyte co-culture assay in the presence or absence of the Smad2 inhibitor SB431542 (Figure 5A). MSCs were treated with SB431542 (10 μM final concentration) 4 h before treatment with TGF-β where relevant. Smad2 inhibition in TGF-β MSC cultures resulted in loss of immunosuppression, with proliferation levels significantly restored to levels similar to unlicensed MSCs. This was demonstrated for both CD4^+^ and CD8^+^ T cell proliferation for more than three generations (Figure 5A). Inhibition of Smad2 signaling in TGF-β MSC wells also significantly reduced the proportion of CD4^+^Foxp3^+^ Tregs present following co-culture (Figure 5A). These results indicate that TGF-β signals through the canonical Smad2/3 pathway in MSCs and that TGF-β MSC-mediated immunosuppression can be attributed to Smad2/3 signaling. We next sought to determine whether the enhanced immunoregulatory characteristics of TGF-β MSCs were cell contact-dependent or -independent. To do this, we performed transwell (TW)-based co-culture assays. Following co-culture, CD4^+^ and CD8^+^ T cell proliferation and frequency of CD4^+^Foxp3^+^ Tregs were analyzed. Direct contact TGF-β MSCs + syngeneic splenocytes served as a positive control (Figure 5B). The results showed that, when co-cultured in the TW system, TGF-β MSCs lost their ability to inhibit proliferation of both CD4^+^ and CD8^+^ T cells, with a significant restoration of proliferation observed for CD4^+^ T cells. Although not significant, a clear trend toward restoration of proliferation for CD8^+^ T cells when compared to direct contact TGF-β MSCs was observed (Figure 5B). Furthermore, levels of Tregs were significantly reduced when TGF-β MSCs were cultured in the TW system, when compared to direct contact TGF-β MSCs (Figure 5B), highlighting the importance of cell-to-cell contact for TGF-β MSC-induced expansion of Tregs. We next wanted to investigate what effects inhibiting CD73 may have on T cell proliferation and Treg expansion. We used the CD73 inhibitor AMP-CP (α,β-Methyleneadenosine 5′-diphosphate sodium salt) in direct co-culture assays. AMP-CP functions by blocking *Nt5e*-mediated adenosine production by preventing the conversion of AMP to adenosine.^66^ Following co-culture, we observed partial restoration of CD4^+^ T cell proliferation and significant restoration of CD8^+^ T cell proliferation in TGF-β MSC wells in which CD73 function was inhibited, compared to TGF-β MSC wells without the inhibitor (Figure 5C). Interestingly, CD73 inhibition had no detectable effect on the frequency of CD4^+^Foxp3^+^ Tregs (Figure 5C). In the context of MSC immunomodulation, PGE2 has been consistently reported to be one of the main mediators of their immunosuppressive function^67^ and, given that we observed a 73-fold increase in *Ptgs2* expression by TGF-β MSCs compared to untreated MSCs (Figure 4D), coupled with a significant increase in PGE2 production following co-culture of TGF-β MSCs with syngeneic lymphocytes (Figure 3B), we investigated what effects blocking of PGE2 signaling would have on T cell proliferation and Treg expansion. We used the PGE2-specific, highly selective EP4 receptor antagonist L-161,982 to achieve this. PGE2 functions by binding to one of four different EP receptors (EP1–EP4), the expression of which is dependent on location, tissue type, cell type, and binding affinity.^68^ We chose an EP4 antagonist in this assay, as it was one of the two high-affinity receptors (the other being EP3) to which PGE2 binds. Binding to EP1 and EP2 requires significantly higher concentrations of PGE2 in order to initiate signaling.^69^ As shown in Figure 5D, inhibition of PGE2 signaling by blocking its binding to EP4 led to a significant restoration of CD8^+^ T cell proliferation and a clear, albeit not statistically significant, restoration of CD4^+^ T cell proliferation in wells containing TGF-β MSCs (Figure 5D). We also observed a significant reduction in the frequency of CD4^+^Foxp3^+^ Tregs when L-161,982 was present (Figure 5D). To confirm the specificity of this approach (i.e., blocking EP4 binding), we performed the same experiment using the selective EP1 receptor antagonist SC-51322 and again examined its effect on TGF-β MSC-induced expansion of Tregs (Figure S4). In contrast to blocking EP4 receptor binding, EP1 antagonism had no discernible effect on the ability of TGF-β MSCs to increase the frequency of CD4^+^Foxp3^+^ Tregs (Figure S4), perhaps suggesting that a PGE2 concentration threshold in the microenvironment is pivotal to effective functioning of TGF-β MSC immunoregulatory mechanisms.

### Systemically Administered TGF-β MSCs Significantly Prolong Corneal Allograft Survival Compared to Their Unlicensed Counterparts

**Figure 6:**
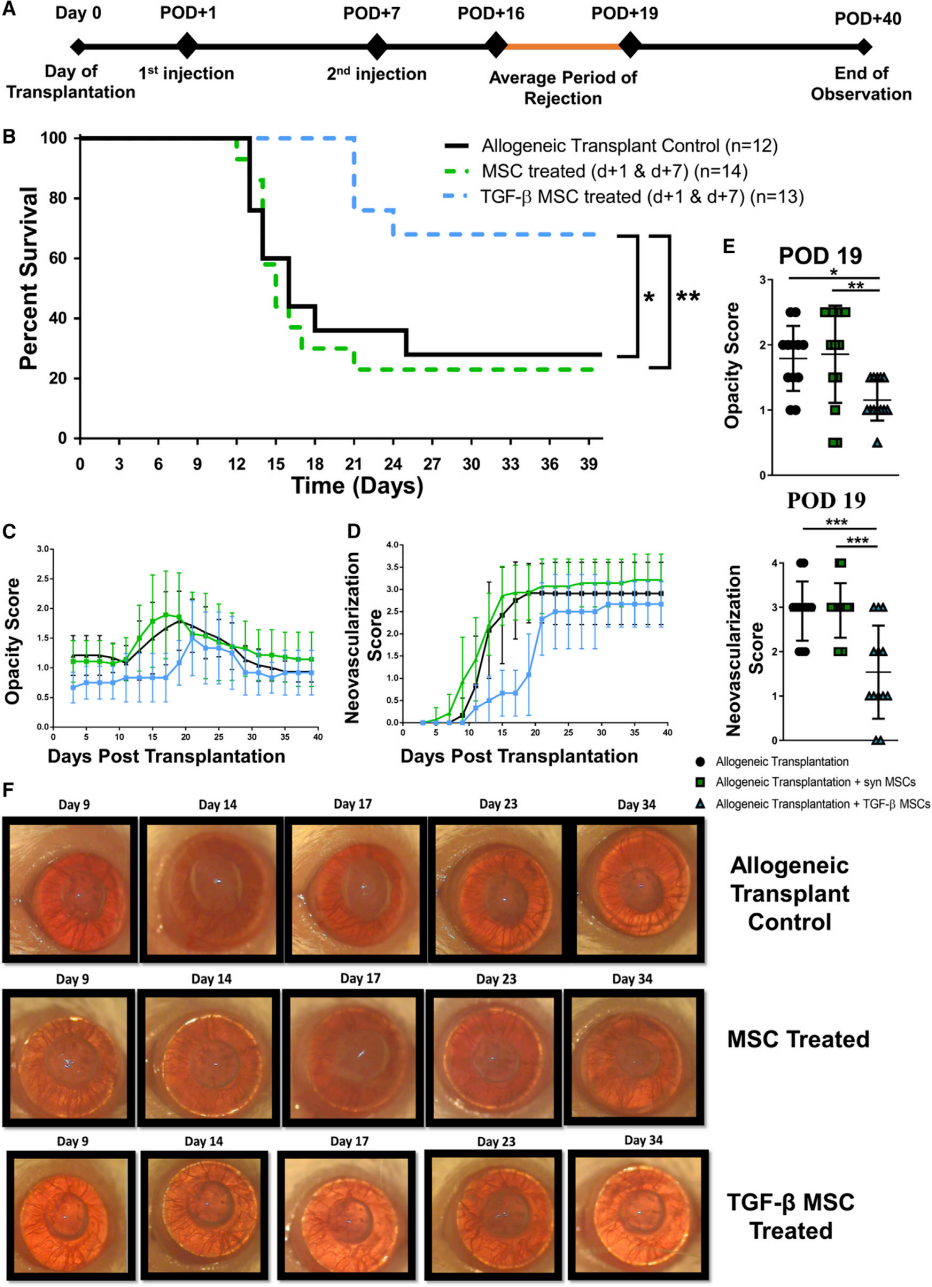
TGF-β MSCs Prolong Corneal Allograft Survival (A) Timeline of events and injection strategy used to assess the ability of MSCs with or without TGF-β pre-activation to prolong corneal allograft survival. Female BALB/c mice served as recipients for female C57BL/6 donor corneas. 1 × 10^6^ untreated or TGF-β MSCs were injected intravenously on post-operative day (POD) 1 and POD 7. Mice were observed every 2–3 days. (B) Kaplan-Meier survival curve analysis of allogeneic transplant controls (black line) (n = 12), corneal allograft + MSCs (dashed green line) (n = 14), and corneal allograft + TGF-β MSCs (dashed blue line) (n = 13). ∗p = 0.05, ∗∗p = 0.01, by log-rank (Mantel-Cox) test. (C and D) Opacity (C) and neovascularization (D) scores up to POD 40 (n = 12–14 with numbers per treatment group the same as in B). (E) Opacity and neovascularization scores were compared between the three groups at POD 19 (corresponding to the average time point of rejection in allogeneic transplant controls) (n = 12–14 with numbers per treatment group the same as in B). (F) Representative light microscope images of corneal transplants taken at multiple time points during the period of observation. Error bars show mean ± SD. ∗p < 0.05, ∗∗p < 0.01, ∗∗∗p < 0.001, by one-way ANOVA, Tukey’s *post hoc* test (n = 12–14).

Based on the striking ability of TGF-β MSCs to induce Tregs with regard to their heightened immunosuppressive ability, we next tested their potential therapeutic efficacy in a fully allogeneic murine model of corneal transplantation. Female BALB/c mice were used as recipients for female C57BL/6 donor corneas. Recipient mice received two intravenous administrations of 1 × 10^6^ untreated or TGF-β MSCs 1 day and 7 days after transplantation. A two-dose post-transplantation strategy was chosen based on the rationale that MSCs, although injected systemically and not locally, would most likely exert a maximum beneficial effect by being present in the system at a time when the innate immune response directed against the allograft was increasing in intensity (post-operative day [POD] 1) and as the adaptive arm of the immune response becomes activated (POD 7). The experimental timeline is depicted in Figure 6A. Our data showed a significant increase in rejection-free survival for mice treated with TGF-β MSCs with 69.2% of recipients accepting their grafts until at least POD 40 (mean survival time [MST], 34.4 ± 8.8 days SD), compared to mice receiving unlicensed MSCs, with only 21.4% of recipients in this group accepting their grafts at the end of the observation period (MST, 20.4 ± 10.9 days SD) (Figure 6B). Opacity score, as the primary indicator of graft rejection, showed a clear decrease in TGF-β MSC-treated recipients (Figure 6C), as did the level of neovascularization, another key clinically relevant parameter commonly used in assessing graft integrity (Figure 6D). We also analyzed opacity and neovascularization levels more specifically at POD 19, a time point falling within the average period of rejection, and observed that TGF-β MSC-treated mice had significantly lower opacity when compared to unlicensed MSC-treated recipients (Figure 6E). TGF-β MSC-treated mice also demonstrated significantly lower neovascularization at the same time point when compared to unlicensed MSC-treated animals (Figure 6E). Light microscope images of corneal allografts from mice treated with TGF-β MSCs remained transparent throughout the observation period, in contrast to unlicensed MSC-treated and allogeneic control transplant recipients (Figure 6F). Overall, the data showed that post-transplant administration of syngeneic TGF-β MSCs significantly prolongs fully MHC-mismatched corneal allograft survival. We next sought to understand the mechanisms of action of the cellular therapy *in vivo*. As unlicensed MSCs showed no therapeutic efficacy, with corneal allograft survival rates comparable to untreated allograft recipients, we focused our *ex vivo* analysis on TGF-β MSC-treated and untreated recipients.

### TGF-β MSCs Significantly Reduce the Levels of Antigen-Presenting Cells (APCs) in Key Model-Specific Organs of Corneal Allograft Recipient Mice at the Average Time Point of Graft Rejection

**Figure 7:**
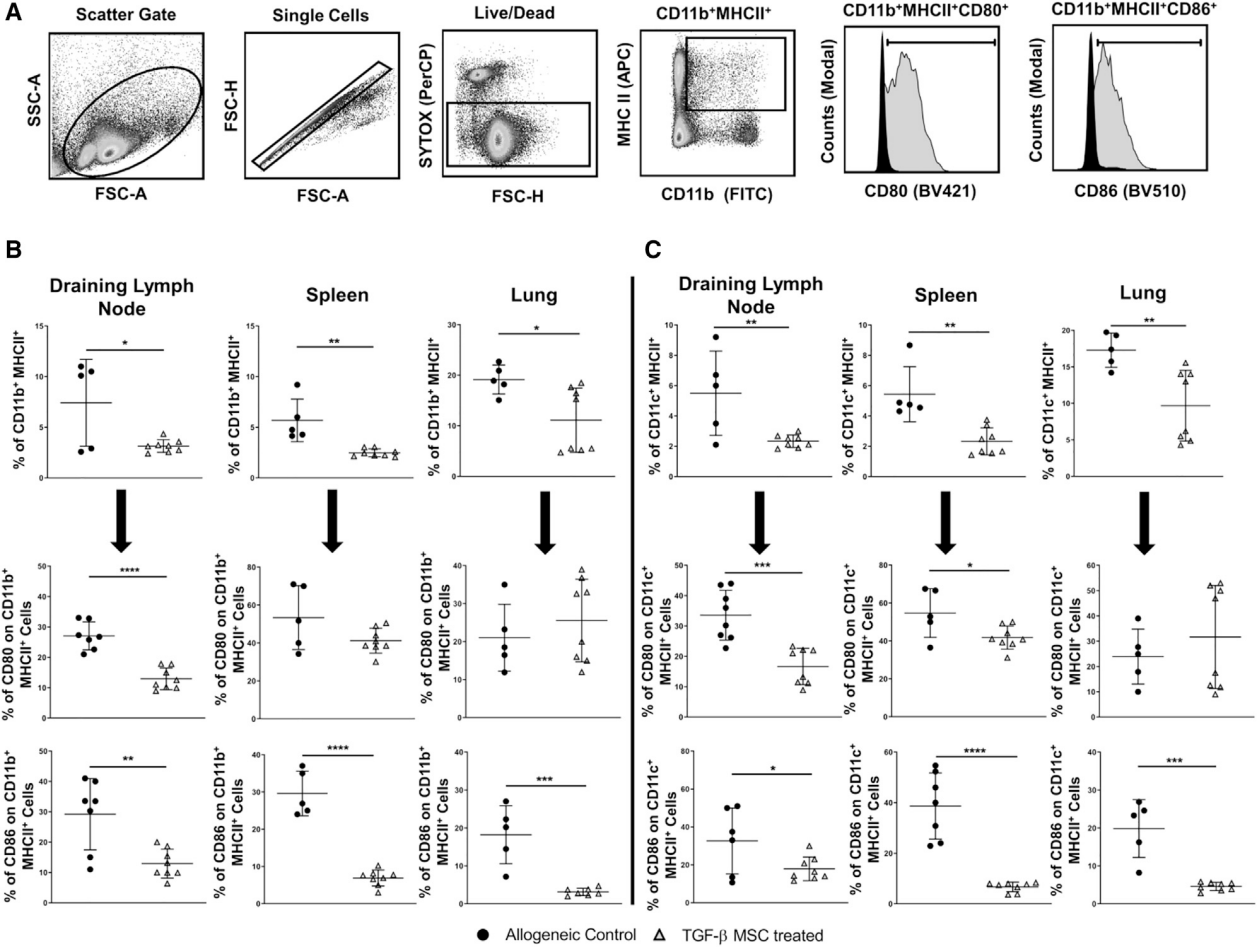
Both the Frequency of and Co-stimulatory Molecule Expression by MPhs and DCs Are Significantly Reduced in Organs of TGF-β MSC-Treated Mice (A) Flow cytometry gating strategy used to select MPhs and DCs. MPhs were selected by using CD11b (FITC) and MHC-II (APC) double positivity. DCs were selected by using CD11c (FITC) and MHC-II (APC) double positivity. CD80 (Brilliant Violet 421 [BV421]) and CD86 (Brilliant Violet 510 [BV510]) were used to analyze the expression of co-stimulatory molecules. (B) Frequency (%) of CD11b^+^MHC-II^+^ MPhs in the dLNs, spleens, and lungs of allogeneic transplant control and TGF-β MSC-treated mice on the average day of rejection. Also shown are CD80 and CD86 expression on CD11b^+^MHC-II^+^ MPhs at the same time point. Error bars show mean ± SD. ∗p < 0.05, ∗∗p < 0.01, ∗∗∗p < 0.001, ∗∗∗∗p < 0.0001 (each individual dot represents a separate animal; n = 4–8). (C) Frequency (%) of CD11c^+^MHC-II^+^ DCs in the dLNs, spleens, and lungs of allogeneic transplant control and TGF-β MSC-treated mice on the average day of rejection. Also shown are CD80 and CD86 expression on CD11c^+^MHC-II^+^ DCs at the same time point. Error bars show mean ± SD. ∗p < 0.05, ∗∗p < 0.01 ∗∗∗p < 0.001 ∗∗∗∗p < 0.0001 (each individual dot represents a separate animal, n = 4–8). A D’Agostino and Pearson omnibus normality test and Shapiro-Wilk normality test were used to determine distribution of data. ROUT testing was used to identify outliers. Parametric unpaired two-tailed Student’s t tests were used for data that were normally distributed. Non-parametric unpaired two-tailed Student’s t tests were used for data that were not normally distributed.

Given the importance of the draining lymph nodes (dLNs) as sites of antigen presentation and T cell activation,^70^ coupled with the well-documented finding that the majority of intravenously administered MSCs become trapped in the lung and are cleared within 24 h,^71^, ^72^, ^73^, ^74^ we analyzed frequencies of both mononuclear phagocytes (MPhs; CD11b^+^) and dendritic cells (DCs; CD11c^+^) in the dLNs and lungs of corneal allograft recipient mice at the average time point of graft rejection. We also analyzed frequencies of these cell types in the spleen, given its crucial role as a secondary lymphoid organ. An example of the gating strategy used to identify CD11b^+^MHC-II^+^ MPhs is shown in Figure 7A. A similar strategy was used for identifying CD11c^+^MHC-II^+^ DCs. Frequency of CD11b^+^MHC-II^+^ MPhs was significantly reduced in all three of the analyzed tissues in mice treated with TGF-β MSCs, compared to untreated allograft recipient mice. Frequencies in the dLNs were 7.14% ± 4% SD compared to 3.14% ± 0.62% SD for TGF-β MSC-treated mice (Figure 7B). Frequencies decreased from 5.67% ± 2.1% SD in the spleens of untreated allograft recipients to 2.47% ± 0.4 SD in the spleens of TGF-β MSC recipients (Figure 7B), and in the lungs they were significantly reduced from 19.16% ± 2.8% SD to 11.10% ± 6.3% SD (Figure 7B). We also analyzed the proportion of CD11b^+^MHC-II^+^ MPhs co-expressing the co-stimulatory molecules CD80 and CD86 and found that the percentage expression of CD80 and CD86 in the dLNs was significantly reduced in TGF-β MSC-treated mice compared to untreated allograft recipients (mean for CD80, 27% ± 4.6% SD in untreated allograft recipients compared to 12.8% ± 3.5% SD in TGF-β MSC-treated mice; mean for CD86, 29.19% ± 11.1% SD in untreated allograft recipients compared to 12.92% ± 4.7% SD in TGF-β MSC-treated mice) (Figure 7B). CD86 expression levels on CD11b^+^MHC-II^+^ MPhs in both the spleens and lungs of TGF-β MSC-treated recipients were also significantly decreased, reducing from 30% ± 5.19% SD in untreated allograft recipients compared to 6.8% ± 5.19% SD in TGF-β MSC-treated mice and 18.2% ± 7.6% SD in untreated allograft recipients compared to 3.14% ± 0.96% SD in TGF-β MSC-treated recipients, respectively.

Highly comparable results were observed when we analyzed frequencies of CD11c^+^MHC-II^+^ DCs in the three tissues. Frequencies in the dLNs were 5.5% ± 2.78% SD compared to 2.33% ± 0.41% SD for TGF-β MSC-treated mice (Figure 7C). Frequencies decreased from 5.43% ± 1.82% SD in the spleens of untreated allograft recipients to 2.32% ± 0.89% SD in the spleens of TGF-β MSC recipients (Figure 7C), and in the lungs they were significantly reduced from 17.28% ± 2.35% SD to 9.68% ± 4.85% SD (Figure 7C). CD80 and CD86 co-stimulatory molecule expression was also significantly decreased on CD11c^+^MHC-II^+^ DCs in both the dLNs and spleens of TGF-β MSC-treated allograft recipients, with CD86 expression, specifically, significantly reduced in lungs of TGF-β-MSC-treated mice (mean for CD80: dLNs, 33.58% ± 8.2% SD in untreated allograft recipients compared to 16.66% ± 5.99% SD in TGF-β MSC-treated mice; spleens, 54.7% ± 12.86% SD in untreated allograft recipients compared to 41.76% ± 6.1% SD in TGF-β MSC-treated mice; mean for CD86: dLNs, 32.61% ± 17.37% SD in untreated allograft recipients compared to 17.9% ± 6.26% SD in TGF-β MSC-treated mice; spleens, 38.68% ± 13.07% SD in untreated allograft recipients compared to 6.7% ± 1.88% SD in TGF-β MSC-treated mice; lungs, 19.84% ± 7.62% SD in untreated allograft recipients compared to 4.58% ± 1.09% SD in TGF-β MSC-treated mice) (Figure 7C). Taken together, these findings indicate that TGF-β-MSC administration has a profound effect on both APC populations. To further validate our *ex vivo* findings, we performed *in vitro* co-culture assays with TGF-β MSCs and bone marrow-derived, LPS + IFN-γ-stimulated macrophages for 72 h (Figure S5A). TGF-β MSCs could significantly decrease the expression of both MHC-I and MHC-II and the co-stimulatory molecule CD80 (Figure S5A). Expression levels of CD86 were not significantly altered. We next analyzed co-culture supernatants for the presence of pro-inflammatory factors. Compared to unlicensed MSCs, co-culture with TGF-β MSCs yielded significantly higher levels of the monocyte chemoattractants CXCL1 and CCL2, but significantly lower levels of the predominantly lymphocyte-specific CCL5 (Figure S5B). IL-1β and TNF-α levels were significantly lower in wells containing TGF-β MSCs compared to unlicensed MSCs (Figures S5B and S5C). Taken together, these results suggest an overall suppression of pro-inflammatory mediators without a concomitant promotion of anti-inflammatory molecule expression.

### TGF-β MSC-Mediated Prolongation of Corneal Allograft Survival Is Associated with Induction of Tregs and Suppression of Activated Effector T Cells

**Figure 8:**
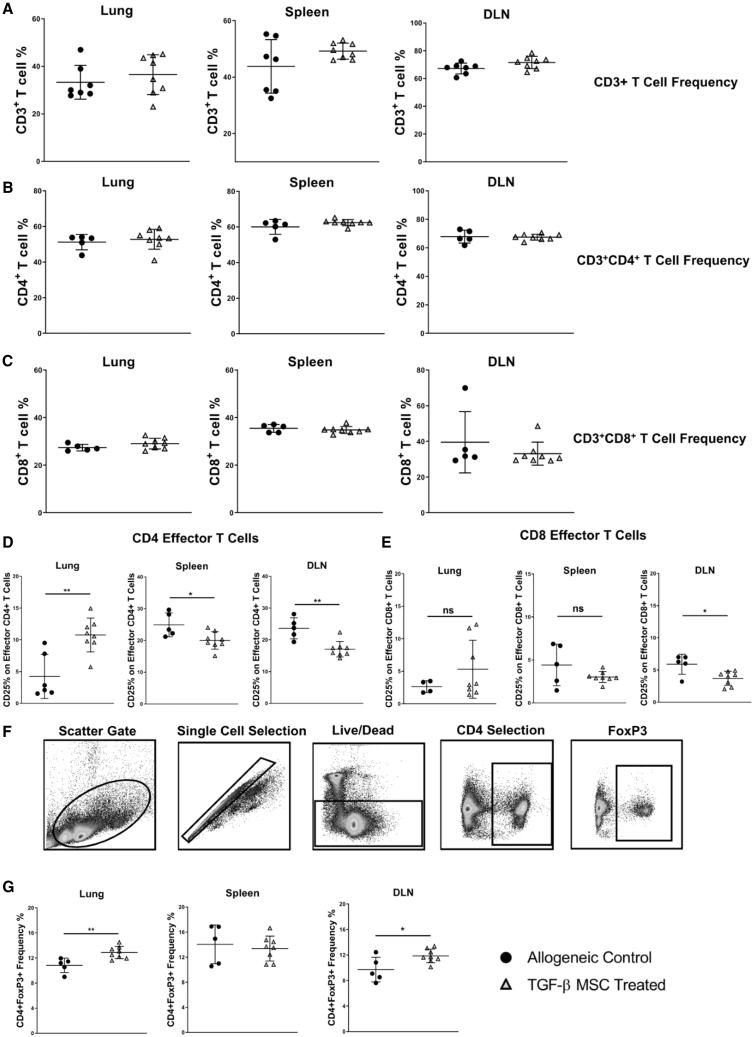
Regulatory T Cell Levels Are Increased in the Lungs and dLNs of Allograft-Recipient Mice following TGF-β MSC Administration (A–C) Total (A) CD3^+^, (B) CD3^+^CD4^+^, and (C) CD3^+^CD8^+^ T cell frequencies in the lungs, spleens, and dLNs of untreated and TGF-β MSC-treated corneal allograft-recipient mice were investigated by flow cytometry at POD 19. (D and E) Frequency (%) of CD3^+^CD4^+^CD44^lo^CD62L^−^CD25^+^ (D) and CD3^+^CD8^+^CD44^lo^CD62L^−^CD25^+^ (E) activated effector T cells in the lungs, spleens, and dLNs of untreated and TGF-β MSC-treated corneal allograft-recipient mice at POD 19 are shown. (F) Flow cytometry gating strategy used to select CD4^+^Foxp3^+^ Tregs. (G) Frequency (%) of CD4^+^Foxp3^+^ Tregs in the lungs, spleens, and dLNs of untreated and TGF-β MSC-treated corneal allograft-recipient mice at POD 19. Error bars show mean ± SD. ∗p < 0.05, ∗∗p < 0.01. A D’Agostino and Pearson omnibus normality test and Shapiro-Wilk normality test were used to determine distribution of data. ROUT testing was used to identify outliers. A parametric unpaired two-tailed Student’s t test was used for data that were normally distributed. A non-parametric unpaired two-tailed Student’s t test was used for data that were not normally distributed (each individual dot represents a separate animal; n = 4–8).

Having established that TGF-β MSCs can effectively modulate the frequency and activation of both MPhs and DCs in multiple organs relevant to both the transplant model and route of MSC administration, we assessed the effects of TGF-β MSCs on key T cell subsets. Initially, we analyzed frequencies of total CD3-expressing T cells to ascertain whether TGF-β MSCs suppress multiple T cell subsets in a broad, non-specific manner. As shown in Figure 8A, however, the proportions of CD3^+^ T cells in lung, spleen, and lymph nodes were largely similar between the untreated allograft recipient and TGF-β MSC-treated groups. Similar results were obtained for total CD4^+^ (Figure 8B) and CD8^+^ (Figure 8C) T cells in each tissue. More comprehensive analysis of specific CD4^+^ and CD8^+^ T cell subtypes revealed a significantly lower proportion of CD4^+^ activated effector T cells (CD3^+^CD4^+^ CD44^lo^CD62L^−^CD25^+^) in both the dLNs and spleens of TGF-β MSC-treated allograft recipients (Figure 8D). The opposite was seen in lungs of these mice with TGF-β MSC-treated recipients having a significantly higher proportion of CD4^+^ activated effector T cells compared to untreated controls (Figure 8D). The contrast between the different tissues could be reflective of an ongoing inflammatory process caused by a high percentage of the MSCs traveling to and remaining in the lungs upon infusion. The same trend was observed for CD8^+^ activated effector T cells; however, differences failed to reach statistical significance in the lungs and spleens of transplanted, treated mice (Figure 8E). Given the importance of Tregs in determining corneal allograft fate by suppression of a potentially destructive graft-oriented immune response, we next analyzed frequencies of Foxp3^+^ Tregs in lungs, spleens, and lymph nodes. The data showed significantly higher frequencies of CD4^+^Foxp3^+^ Tregs in both the lungs (mean, 12.87% ± 0.98% SD) and dLNs (11.87% ± 1.08% SD) of TGF-β MSC allograft recipients, compared to untreated controls (10.83% ± 1.14% SD for lungs and 9.71% ± 1.93% SD for dLNs) (Figure 8G; see Figure 8F for gating strategy). Treg levels were comparable in the spleens of both groups of allograft recipients. Taken together, our results show that TGF-β MSCs have the ability to inhibit potentially graft-destroying activated effector T cells, which is associated with the expansion of graft-protecting Tregs.

## Discussion

The pre-activation or licensing of MSCs before *in vivo* administration has become a common strategy to potentially improve the therapeutic efficacy of the cells.^13^^,^^19^^,^^48^^,^^75^, ^76^, ^77^ In this study, we describe a novel licensing strategy for MSCs with enhanced potential for Treg induction. Furthermore, TGF-β licensing leads to a significant reduction of MHC-I that may have important implications for modulating allogeneic immune responses in the setting of organ transplantation.^78^ Allogeneic cell therapies hold great promise for the treatment of various inflammatory conditions, such as type 1 diabetes (T1D),^79^ rheumatoid arthritis,^80^ and uveitis^81^. The finding that TGF-β suppresses MHC-I expression on MSCs may be particularly relevant in the context of allogeneic cell therapies to prevent rejection and warrants further investigation.

Perhaps of most relevance to our current study, as it relates to modulating MSC immunosuppressive capacity via TGF-β1 pre-treatment, Xu et al.^82^ showed that, instead of increasing the suppressive capacity of MSCs following TGF-β1 exposure, the cells actually became less suppressive. It is important, however, to highlight crucial differences in assay design between the two studies. For example, in our assays, MSCs were treated directly with 50 ng/mL of TGF-β1 for 72 h, washed extensively to ensure removal of exogenous TGF-β1, and added to the co-culture assay at the same time as addition of splenocytes. In contrast, the study by Xu et al.^82^ reported seeding MSCs alone 24 h before simultaneous addition of splenocytes and anti-CD3/CD28 polyclonal stimulation beads. Furthermore, differing concentrations of TGF-β1 were added to these wells at 0 and 24 h after splenocyte addition. Our TGF-β1 licensing regime ensured the absence of TGF-β1 itself upon initiation of the co-culture assay (see Figure S2A). Therefore, the effects reported in our study (e.g., on T cell proliferation, activation, Treg expansion, and *in vivo* efficacy) are as a result of the altered phenotype conferred upon the MSCs by TGF-β treatment.

Our finding that TGF-β MSCs co-cultured with syngeneic splenocytes induces a Treg population with elevated CD73 expression may have particular relevance given the significant prolongation of allograft survival observed. Tregs have long been established as having an important role to play in the prolongation of graft survival, including corneal allograft survival,^6^^,^^83^, ^84^, ^85^, ^86^ and the induction of Tregs has been attributed to the immunomodulatory molecule PGE2 in certain contexts.^77^^,^^87^ One such example of how PGE2 can do this was shown by Baratelli et al.^88^ who demonstrated that PGE2, by modulating the expression of the transcription factor FOXP3, could induce a Treg phenotype from CD4^+^CD25^−^ T cells. Interestingly, Mandapathil et al.^89^ could demonstrate that human Tregs co-expressing the ectonucleotidases CD39 and CD73, as well as *Cox-2*, whose activity leads to the production of immunomodulatory adenosine and PGE2, respectively, had higher suppressive capacity and suggested that because both molecules use the same intracellular signaling pathway, their cooperation likely contributed to Treg-mediated suppression.^89^ Indeed, we observed that TGF-β-licensed MSCs expressed significantly higher levels of *Ptgs2* and secreted significantly higher levels of PGE2 compared to their unlicensed counterparts. The importance of TGF-β MSC-derived PGE2 production was further highlighted by the finding that inhibition of PGE2/EP4 receptor binding reversed the expansion of Tregs. Furthermore, in the context of Treg expansion, PGE2 has been reported to function in a cell contact-dependent manner.^90^ In line with our data, removal of cell-to-cell contact using TW inserts completely reversed the Treg expansion observed in direct contact co-cultures. These findings suggest that, of the combined *in vitro* and *in vivo* putative mediators of the *in vivo* therapeutic effects, PGE2-mediated Treg induction is the principal mechanism.

The importance of the dLNs in corneal allograft rejection has been clearly demonstrated by Yamagami et al.^91^^,^^92^ following removal of the dLNs in both low- and high-risk murine models of corneal transplantation. Removal of the dLNs prevented graft rejection in both models, demonstrating the importance of the initial antigen presentation phase and the subsequent expansion of alloreactive effector T lymphocytes. For this reason, we investigated whether TGF-β MSCs could indirectly modulate immune cell populations in the dLNs of allograft recipients. The results showed that, similar to the observations in the lungs of TGF-β MSC-treated animals, frequencies of CD3^+^CD4^+^Foxp3^+^ Tregs were also significantly increased in the dLNs. The increased frequency of Tregs may contribute to the suppression of the alloreactive T lymphocyte response directly or indirectly by modulating APCs in the dLNs. This is supported by the observed significant decreases in both DC and MPh frequencies in the dLNs, coupled with significant decreases in the frequencies of both CD80 and CD86 expressing MHC-II^+^ DCs and MPhs (Figure 7).

Another potential application of this MSC licensing strategy (i.e., TGF-β1 pre-conditioning) worth considering is in the area of polyclonal Treg expansion. As Treg frequency in peripheral blood, for example, is low (<1% of leukocytes),^93^ Tregs usually need to be expanded *in vitro* to obtain sufficient numbers for clinical applications. As reviewed by MacDonald et al.,^94^ the only clinical manufacturing protocol for Treg expansion published to date utilizing TGF-β does so by adding it as a supplement.^95^ In the present study, we show that, even at relatively conservative MSC/splenocyte ratios (1:10), TGF-β pre-conditioning of MSCs can increase Treg numbers by 43.35% versus polyclonally stimulated splenocytes and 26.24% versus untreated MSCs (Figure 2E). Moreover, these expanded Tregs have increased suppressive ability compared to untreated MSCs, as they express higher levels of both CD73 (Figure 2F) and PD-L1 (Figure 2G). While other factors must of course be considered such as use of antibody-coated stimulation beads (as used herein) or artificial APCs for stimulation purposes, supplementation of culture media with cytokines (e.g., IL-2) or immunosuppressive drugs (e.g., rapamycin), this method of Treg expansion utilizing licensed MSCs could hold promise with further optimization for this specific purpose.

Considering the well-documented complex role of TGF-β in tumor progression,^96^^,^^97^ the combination of immunosuppressive effects of TGF-β licensing on MSCs may also uncover targetable signaling mechanisms prevalent in certain stromal cell-dense solid tumor microenvironments^98^. Treg expansion in tumors is associated with poor prognosis,^99^^,^^100^ and these findings may open new therapeutic opportunities for targeting TGF-β-induced immunosuppressive effects, through Treg expansion, in the tumor microenvironment.

In conclusion, our results show that pre-treatment of MSCs with TGF-β generates a novel, unique MSC phenotype as characterized by their increased expression/secretion of immunomodulatory molecules and their enhanced capacity to induce regulatory cell populations both *in vitro* and *in vivo*. This unique footprint of TGF-β-treated MSCs may have important implications for treatment of many clinical indication/pathologies, including transplant rejection, autoimmune diseases, and graft-versus-host disease.^101^^,^^102^ TGF-β licensing of MSCs represents a novel strategy that, in addition to suppressing T cell proliferation, has a unique and potent ability to expand Tregs. Tregs have been shown to be essential in suppressing pathological T cell responses and are emerging as a potential cell-based therapy for treatment of systemic lupus erythematosus, T1D, and inflammatory bowel disease.^103^

## Materials and Methods

### Animals and Corneal Transplantation

All procedures performed on mice were approved by the Animals Care Research Ethics Committee of the National University of Ireland, Galway (NUIG) and conducted under individual and project authorization licenses from the Health Products Regulatory Authority (HPRA) of Ireland. All animals were housed and cared for under standard operating procedures of the Animal Facility at the Biomedical Sciences Biological Resource Unit, NUIG. A fully allogeneic MHC class I/II disparate cornea transplant model was used for these studies. Female C57BL/6 (H-2^b^) mice served as corneal graft donors, and female BALB/c (H-2^d^) or transgenic BALB/c C.Cg-*Foxp3*^*tm2Tch*^/J (H-2^d^) mice served as recipients. All animals were aged between 8 and14 weeks old and obtained from Envigo Laboratories (Oxon, UK) or The Jackson Laboratory (Bar Harbor, ME, USA) and housed with food and water *ad libitum*. Orthotopic corneal transplantation was performed as follows. For induction of anesthesia, animals were placed in an anesthesia box connected to an isoflurane vaporizer and pre-filled with a mixture of oxygen and isoflurane (5% anesthetic in 2 L/min medical oxygen; BOC Gases, Galway, Ireland). For surgical anesthesia, a mixture of ketamine (90 mg/kg) and xylazine (7.5 mg/kg) was injected intraperitoneally. Deep anesthesia was achieved when limb withdrawal and eye reflexes were abolished. Depth of anesthesia was monitored by the breathing pattern of the animals. 1% tetracaine (Chauvin Pharmaceuticals, Kingston upon Thames, UK) was administered as a local anesthetic, and 1% atropine sulfate, 1% tropicamide, and 2.5% phenylephrine hydrochloride (all Chauvin Pharmaceuticals) were administered for pupil dilation. A 1.5-mm graft bed was prepared and a 2-mm donor graft was sutured in place with a continuous looped 11-0 Ethilon suture (Ethicon, Livingston, Scotland). Antibiotic ointment containing chloramphenicol was applied to the graft. Irrigation of the corneal tissue was achieved by application of balanced salt solution (BSS) (Alcon, Hemel Hempstead, UK). Eyelids were sutured closed to prevent the animals from scratching the graft and were removed 2 days after surgery. Corneal sutures were removed 7 days after surgery. Graft opacity as the primary indicator of rejection was scored every 2–3 days using a Leica operating microscope at ×25 magnification and graded on a scale of 0–3, with 0 being a completely transparent cornea and 3 corresponding to complete corneal opacity, with the anterior chamber not visible. A graft was considered to be rejected when an opacity score of 2 was recorded on 2 consecutive days or one score of 2.5 or above.^104^ Animals with surgical complications were excluded.

### Mouse MSC Isolation, Culture, and Characterization

Mice were euthanized by CO_2_ inhalation and the femur and tibia were removed, cleaned of connective tissue, and placed in MSC medium consisting of MEM-α (BioSciences, Dublin, Ireland) supplemented with 10% heat-inactivated fetal bovine serum (FBS) (Thermo Fisher Scientific, Dublin, Ireland), 10% equine serum (Thermo Fisher Scientific), and 1% penicillin/streptomycin (Sigma-Aldrich, Wicklow, Ireland). The heat-inactivated FBS was pre-screened to ensure it supported MSC growth and differentiation. The epiphysis of each bone was cut, and cells were flushed out with culture medium using a 30.5G needle. Clumps were removed by filtering through a 70-μm cell strainer (Thermo Fisher Scientific). Cells were then centrifuged at 400 × *g* for 5 min, re-suspended in 25 mL of culture medium, and plated at a density of 9 × 10^5^ cells/cm^2^ in a T175 flask (Sarstedt, Wexford, Ireland). Cells were incubated at 37°C, 21% O_2_, 5% CO_2_ (normoxia) or 37°C, 5% O_2_, 5% CO_2_ (hypoxia) and non-adherent cells were removed 24 h later. Medium was changed every 3–4 days and cells were passaged at 85% confluency. MSCs were cultured and used in subsequent experiments up to passage 10 (P10). Successful differentiation to osteogenic and adipogenic cell lineages was confirmed prior to downstream use (Figure S1). Furthermore, cell surface characterization for established markers was carried out by flow cytometry (see Table S1 for antibody catalog numbers).

### TGF-β Licensing of MSCs

BALB/c MSCs were seeded at a density of 50,000 cells/mL of MSC medium in a T175 flask (Sarstedt). 12 h after seeding, medium was removed and replaced with mouse MSC medium containing recombinant mouse TGF-β at a concentration of 50 ng/mL (Bio-Techne, Abingdon, UK) (see Table S2 for additional information). Cells were then placed in a humidified tissue culture incubator at 37°C, 5% CO_2_. 72 h later, medium was removed and cells were washed twice with Dulbecco’s phosphate-buffered saline (DPBS) (Thermo Fisher Scientific). To detach the cells from the flasks, 5 mL/T175 flask of 0.25% trypsin (Sigma-Aldrich) was added and cells were incubated for 3 min at 37°C, 5% CO_2_. Trypsin was neutralized by adding twice the volume of serum-containing medium. Cells were then centrifuged at 400 × *g*, washed twice with DPBS, and counted before use in subsequent experiments.

### Intravenous Administration of MSCs

MSCs were isolated and expanded from BALB/c mice as described above and, where appropriate, were pre-conditioned with TGF-β1 or left untreated. MSCs were washed three times with DPBS and filtered through a 40-μm filter (Thermo Fisher Scientific) before administration. Mice were placed into a cylindrical restraining device and intravenously administered 1 × 10^6^ MSCs or TGF-β MSCs in 100 μL of PBS through the lateral tail vein using a 30G needle.

### Tissue Separation, Cell Isolation, and Flow Cytometry

Lungs were digested by first cutting the tissue into pieces approximately 1 mm in size, followed by digestion by incubating in Hanks’ balanced salt solution (HBSS) (Thermo Fisher Scientific) containing collagenase IV (200 U/mL) (Thermo Fisher Scientific) and DNase I (200 U/mL) (Sigma-Aldrich) at 37°C for 2 h at 150 rpm. Single-cell suspensions of the lymph node, spleen, and digested lung were prepared by gentle mashing of the organs through a 40-μm cell strainer (Thermo Fisher Scientific) in 6-cm Petri dishes (Sarstedt) containing 5 mL of DPBS. The single-cell suspensions were then centrifuged at 800 × *g* for 5 min. The lymph node cells were washed in DPBS and counted using a hemocytometer. The spleen and lung cells were re-suspended in ACK lysis buffer (distilled water, 0.15 M NH_4_Cl, 10 mM KHCO_3_, 0.1 mM sodium EDTA) and incubated on ice for 5 min. The reactions were stopped by adding complete medium consisting of RPMI 1640 (Thermo Fisher Scientific) supplemented with 10% heat-inactivated FBS, 1% sodium pyruvate (1 mmol/L), 1% non-essential amino acids (0.1 mmol/L), 1% l-glutamine (2 mmol/L), 1% penicillin (100 U/mL)/streptomycin (100 μg/mL), and 0.01% β-mercaptoethanol (55 μmol/L) (all from Sigma-Aldrich). Cells were centrifuged at 800 × g for 5 min, washed, and re-suspended in DPBS and counted.

For flow cytometric analysis, 1 × 10^5^ cells/sample were stained with the following anti-mouse antibodies diluted in fluorescence-activated cell sorting (FACS) buffer (DPBS supplemented with 1% FBS and 0.05% sodium azide): CD3, CD4, CD8, CD25, CD44, CD45.2, CD62L, CD11b, CD11c, MHC-I, MHC-II, CD80, CD86, and PD-L1 (all from Biolegend) (see Table S1 for antibody catalog numbers) and with the cell viability dyes SYTOX Blue or SYTOX AADvanced (Thermo Fisher Scientific). Samples were analyzed using a BD FACSCanto II flow cytometer (BD Biosciences, San Jose, CA, USA). Flow cytometry data were analyzed using FlowJo analysis software version 10 (Tree Star, Ashland, OR, USA).

### *In Vitro* Co-culture Assays

#### Macrophage Isolation and MSC/Macrophage Co-culture

Femurs and tibias were removed from BALB/c mice, cleaned of connective tissue, and placed in macrophage culture medium. Macrophage medium consisted of 65% complete medium and 35% L929-conditioned medium. Complete medium included RPMI 1640 (Thermo Fisher Scientific) supplemented with 10% heat-inactivated FBS, 1% sodium pyruvate (1 mmol/L), 1% non-essential amino acids (0.1 mmol/L), 1% l-glutamine (2 mmol/L), 1% penicillin (100 U/mL)/streptomycin (100 μg/mL), and 0.01% β-mercaptoethanol (55 μmol/L) (all Sigma-Aldrich). L929-conditioned medium included DMEM medium (Thermo Fisher Scientific), 10% heat-inactivated FBS, and 1% penicillin (100 U/mL)/streptomycin (100 μg/mL) (all Sigma-Aldrich) collected from 3-day cultures of L929 cells, which produce macrophage-colony stimulating factor (M-CSF). The epiphysis of each bone was cut and cells were flushed out with macrophage medium using a 30.5G needle. Clumps were removed by filtering through a 70-μm mesh filter (Thermo Fisher Scientific). Cells were then centrifuged at 400 × g for 5 min, red blood cells were removed using ACK lysis buffer, and the cells were centrifuged at 400 × *g* for 5 min. The cells were re-suspended at a concentration of 2.25 × 10^6^ cells/mL in macrophage medium and plated at a density of 4.5 × 10^6^ per well of a six-well plate. Cells were incubated at 37°C, 5% CO_2_, changing the medium every 2 days. This process was repeated for 6 days. Trypsin was added for 10–12 min at 37°C to detach the macrophages from the plates. Twice the volume of complete medium was added to neutralize the trypsin. If stimulated macrophages were required, IFN-γ (100 U/mL) (see Table S2 for additional information) was added on day 6 for 24 h followed by LPS (10 ng/mL) (see Table S2 for additional information) stimulation for 4 h. For MSC/macrophage co-culture assays, untreated or TGF-β MSCs were added to wells of macrophages at a ratio of 1:5 MSCs/macrophages.

#### Splenocyte Isolation and MSC/Splenocyte Co-culture

For lymphocyte proliferation assays, lymph nodes and spleens were isolated from BALB/c mice and single-cell suspensions were prepared as described above. Cells were re-suspended in complete medium (RPMI 1640 [Thermo Fisher Scientific] supplemented with 10% heat-inactivated FBS, 1% sodium pyruvate [1 mmol/L], 1% non-essential amino acids [0.1 mmol/L], 1% l-glutamine [2 mmol/L], 1% penicillin (100 U/mL)/streptomycin (100 μg/mL), and 0.01% β-mercaptoethanol (55 μmol/L) [all Sigma-Aldrich]). The cells were labeled with a CellTrace Violet cell proliferation kit (Thermo Fisher Scientific) according to the manufacturer’s instructions and seeded in 96-well plates at a concentration of 2 × 10^5^ cells/100 μL of complete medium with or without anti-mouse anti-CD3/CD28 Dynabeads (Thermo Fisher Scientific) at a ratio of 1:4 (beads/lymphocytes). In relevant experiments, untreated or TGF-β MSCs were added to wells of lymphocytes at a concentration of 2 × 10^4^ cells/50 μL (ratio of 1:10 MSCs/lymphocytes) of mouse MSC medium.

For Smad2 inhibition, SB431542 (Cell Signaling Technology, Danvers, MA, USA, catalog no. 14775S) was used at a concentration of 10 μM 2 h prior to treatment with TGF-β. To inhibit CD73 activity, AMP-CP (Sigma-Aldrich, catalog no. M3763) was added to T lymphocyte co-cultures at a final concentration of 100 μM. The selective EP1 antagonist (SC-51322) and selective EP4 antagonist (L-161,982) (both Cayman Chemical, Ann Arbor, MI, USA) (see Table S2 for additional information) were used in T lymphocyte co-cultures to assay the importance of EP/PGE2 signaling. Both the EP1 and EP4 antagonists were used at a final concentration of 1 mM.

### ELISA and Griess Assay for NO Quantification

sTNFR1, sTNFR2, TNF-α, IFN-γ, granzyme B, IL-6, IL-12, IL-1β, CXCL1, CCL2, and CCL5 were detected in culture supernatants from either T lymphocyte or macrophage co-culture assays by magnetic Luminex assays (R&D Systems, Biotechne, Abingdon, UK) (see Table S2 for additional information) according to the manufacturer’s instructions. TNF-α and IL-10 were analyzed in culture supernatants from macrophage co-culture assays using individual Ready-SET-Go! ELISA kits (Affymetrix/eBioscience, Thermo Fisher Scientific) according to the manufacturer’s instructions. PGE2 was detected using an individual PGE2 assay (R&D Systems, Biotechne). TGF-β1 was detected using Ready-SET-Go! ELISA kits (Affymetrix/eBioscience, Thermo Fisher Scientific) according to the manufacturer’s instructions (see Table S2 for additional information on specific ELISA kits).

NO concentration was quantified in culture supernatants by a Griess assay (see Murphy et al.^24^ for details). 100 μL of supernatant was combined with an equal volume of Griess reagent (composed of 1% sulfanilamide and 0.1% *N*-1-(naphthyl)ethylenediamine dihydrochloride in 2.5% H_3_PO_4_) (Abcam, Cambridge, UK) in a 96-well flat bottom plate. Absorbance was measured at 540 nm on a plate reader (PerkinElmer, Ireland).

### RNA-Seq Analysis

RNA-seq and data analysis were outsourced to Arraystar (USA). Briefly, 1–2 μg of total RNA was enriched by oligo(dT) magnetic beads (rRNA removal) and sequencing libraries were prepared. RNA sequencing was performed on the Illumina platform. Raw paired-end RNA sequenced data, contained in FASTQ formatted files, were quality assessed using FASTQC v.011.5.^105^ Subsequent downstream processing and analysis followed both alignment and alignment-free approaches. For the alignment approach, Illumina 5′ and 3′ adapters were trimmed from the sequenced reads using Cutadapt v.1.14.^106^ Trimmed reads were aligned to the Ensembl mm10 reference genome using Hisat2 v.2.0.5.^107^ Transcript abundances for each sample were estimated using StringTie v.1.3.1c.^108^ FPKM Fragments per kilobase of transcript per million mapped reads (FPKM) values were calculated and differential expression (DE) analysis was performed with the R/Bioconductor package Ballgown v2.8.4.^109^ Fold change (cutoff 1.5), p value (≤0.05), and FPKM (≥0.5 mean in one group) were used to filter differentially expressed genes. For the alignment-free approach, Illumina adapters were trimmed using FASTP v.0.19.7.^110^ Transcript quantification was estimated using Salmon v0.8.1.^111^ In this study, an index of the Ensembl mm10 reference transcriptome (built-in Salmon) was used for quasi-mapping of reads with parameters set to automatically detect the library type and correct for GC bias. Mapping rates were assessed for quality of mapping and possible contamination. Transcript-level abundance estimates were summarized to gene-level count data using the R/Bioconductor package Tximport v1.10.1.^112^ Independent filtering of low abundant features (counts of <50 across all samples) was performed to filter out noise and improve sensitivity. Differential expression analysis was conducted using the R/Bioconductor package DESeq2.^113^ Fold change (cutoff 2) and adjusted p (≤0.0001) were used for filtering differentially expressed genes. Gene set enrichment analysis (GSEA)^114^ was performed with differentially expressed genes. The R/Cran package Pheatmap v1.0.12^115^ was used to construct a heatmap of most variable genes, and the R/Bioconductor package EnhancedVolcano v.1.0.1^116^ was used to construct a volcano plot. All data processing, analysis, and graphic generation were performed using R v3.4.1, Python v2.7, or shell environment.

### Statistical Analysis

All experiments were repeated at least three times independently, with two-three technical replicates included per experiment. All statistical analysis was performed using GraphPad Prism software Version 8 (La Jolla, CA, USA). Data were assessed for normal distribution using the Kolmogrov-Smirnov test. Comparisons between two groups were analyzed by Student’s t test. One-way ANOVA was used to analyze results from *in vitro* and *in vivo* experiments containing three or more groups, followed by Tukey’s multiple comparison post-test. Data are presented as mean ± SD. Kaplan-Meier survival curves with log-rank (Mantel Cox) test were used for analysis of allograft survival. Differences were considered significant if p ≤0.05.

## Author Contributions

Substantial contributions to the conception or design of the work (K.L., O.T., N.M., P.L., A.E.R., and T.R.) or the acquisition (K.L., O.T., X.C., N.M., P.L., M.N.I., E.D., L.W., G.O’M. A.E.R., and T.R.), analysis (K.L., O.T., X.C., N.M., P.L., M.N.I., E.D., S.M., G.O’M. A.E.R., and T.R.), or interpretation of data for the work (K.L., O.T., X.C., N.M., P.L., M.N.I., E.D., S.M., M.D.G., G.O’M. A.E.R., and T.R.). Drafting the work or revising it critically for important intellectual content (K.L., O.T., X.C., N.M., P.L., M.N.I., E.D., M.D.G., L.W., S.M., G.O’M. A.E.R., and T.R.), final approval of the version to be published (K.L., O.T., X.C., N.M., P.L., M.N.I., E.D., M.D.G., L.W., S.M., G.O’M. A.E.R., and T.R.), and agreement to be accountable for all aspects of the work in ensuring that questions related to the accuracy or integrity of any part of the work are appropriately investigated and resolved (K.L., O.T., X.C., N.M., P.L., M.N.I., E.D., M.D.G., L.W., S.M., G.O’M. A.E.R., and T.R.).

## Conflicts of Interest

The authors declare no competing interests.

## Supplemental Information

Document S1. Figures S1–S5, Tables S1–S4, and Supplemental Materials and Methods

Document S2. Article plus Supplemental Information
