# Supplementary material for: TGF-β1-Licensed Murine MSCs Show Superior Therapeutic Efficacy in Modulating Corneal Allograft Immune Rejection In Vivo
Source: Mol Ther. 2020 May 30;28(9):2023–43. doi: 10.1016/j.ymthe.2020.05.023 (PMC7474271; doi:10.1016/j.ymthe.2020.05.023)
Supplement: Document S1. Figures S1–S5, Tables S1–S4, and Supplemental Materials and Methods [file mmc1.pdf]

YMTHE, Volume *28*

Supplemental Information

TGF-β1-Licensed Murine MSCs Show Superior Therapeutic Efficacy in Modulating Corneal Allograft Immune Rejection *In Vivo*

Kevin Lynch, Oliver Treacy, Xizhe Chen, Nick Murphy, Paul Lohan, Md Nahidul Islam, Ellen Donohoe, Matthew D. Griffin, Luke Watson, Steven McLoughlin, Grace O'Malley, Aideen E. Ryan, and Thomas Ritter

Supplemental Material

Supplemental Figures

Supplemental Figure 1

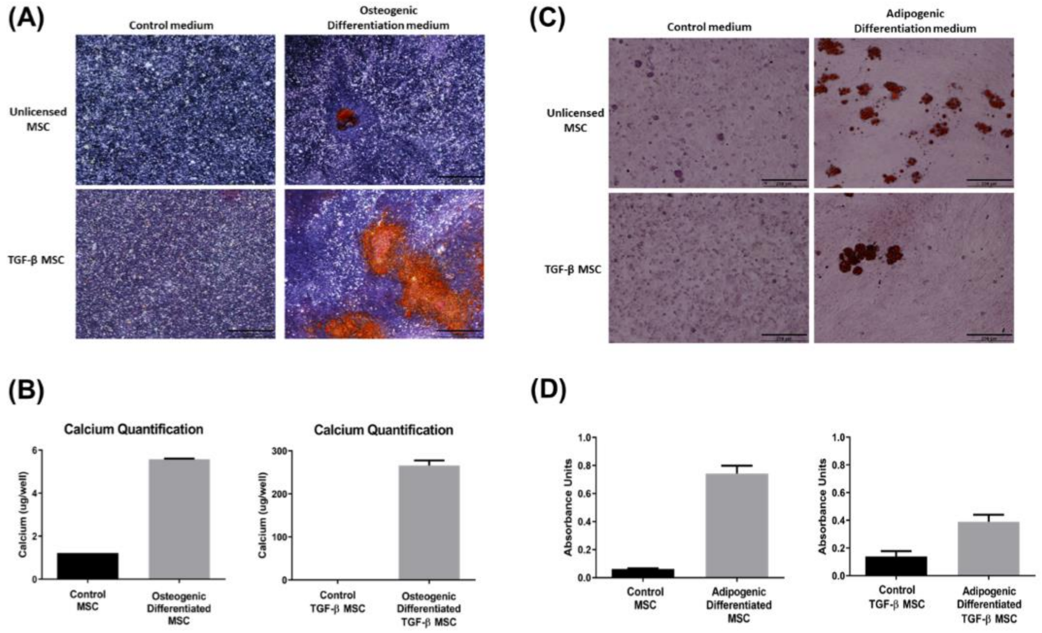

Supplemental Figure 2

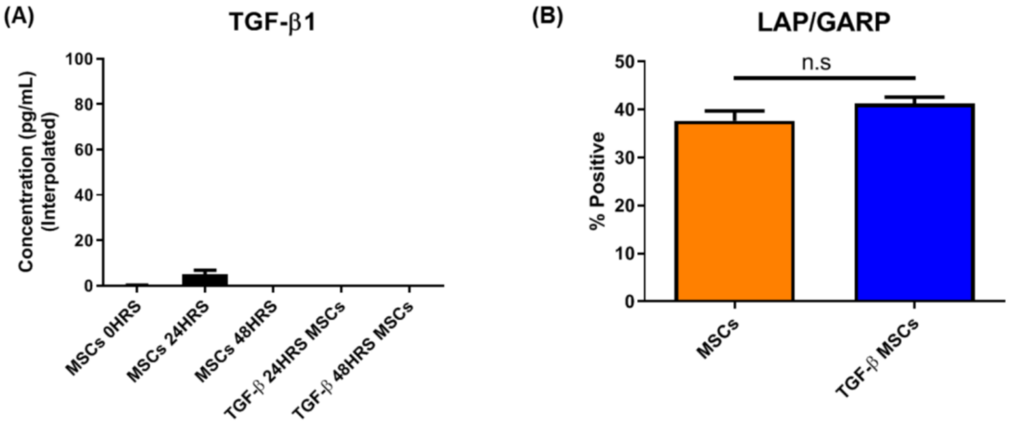

Supplemental Figure 3

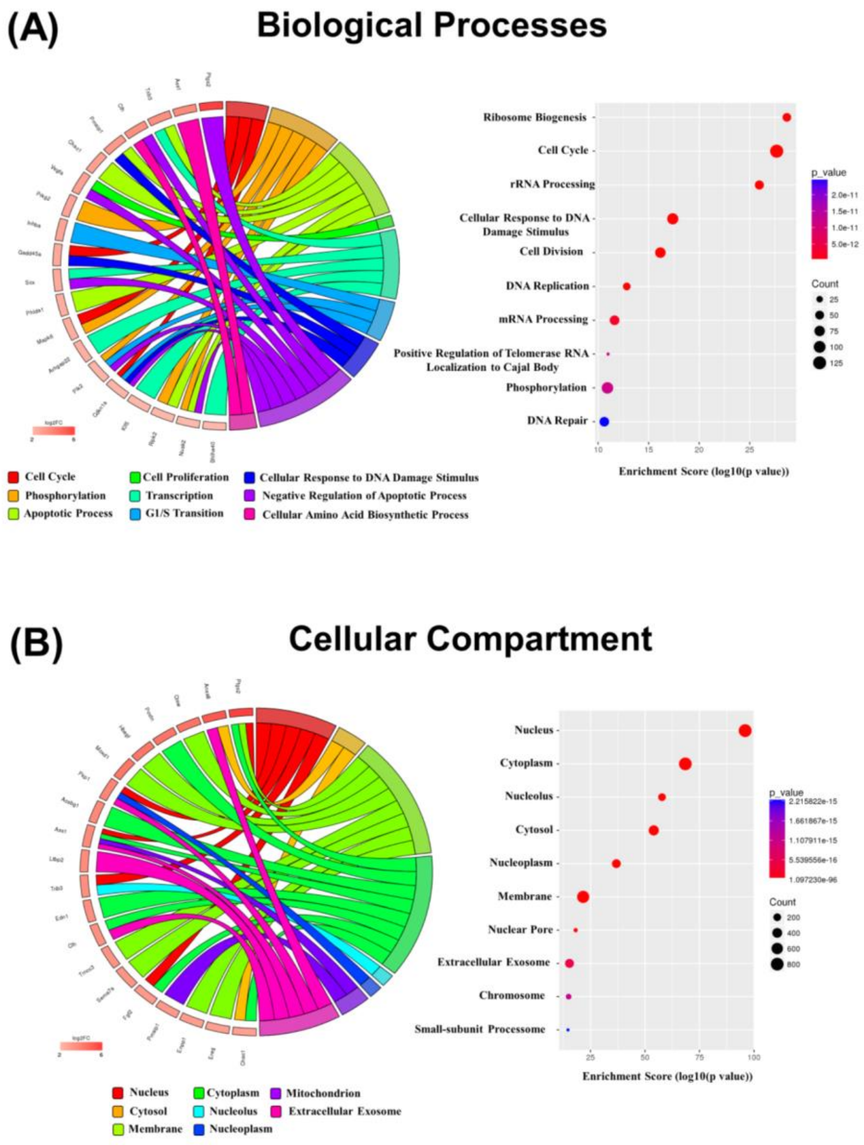

Supplemental Figure 4

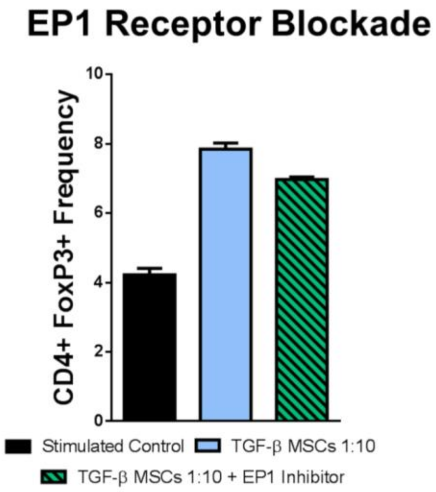

Supplemental Figure 5

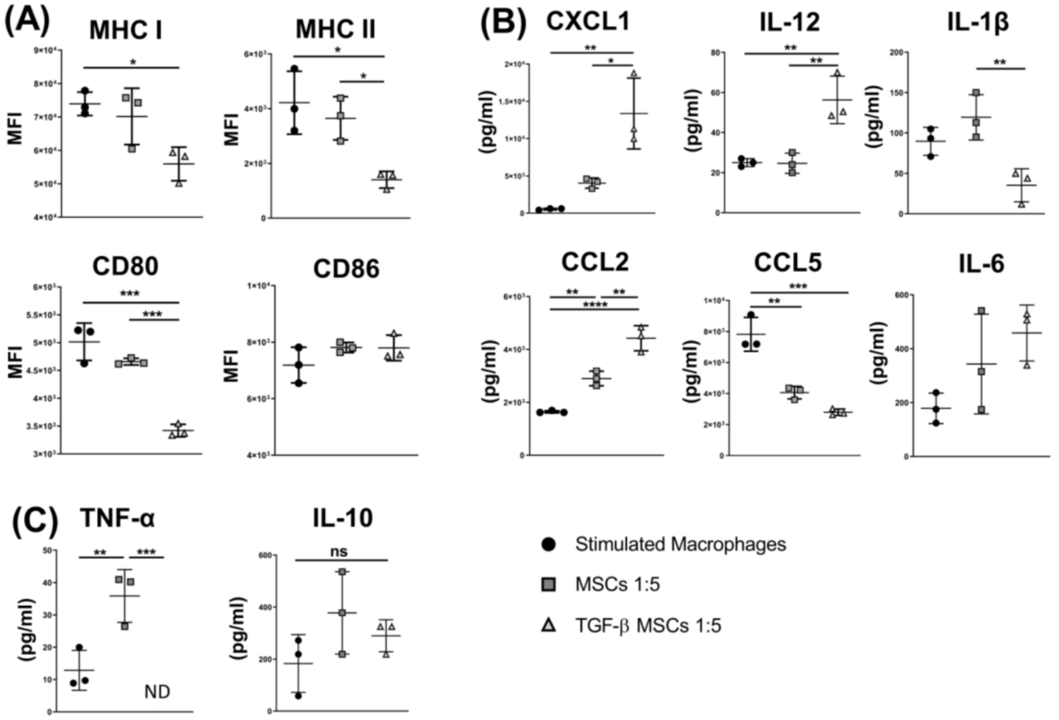

Supplemental Figure legends

Supplemental Figure 1. Osteogenic and adipogenic differentiation of MSCs and TGF-β MSCs. Balb/c MSCs were cultured in specific osteogenic or adipogenic differentiation medium. (A) Brightfield microscopy images of Alizarin Red stained control and osteogenic differentiated MSCs and TGF-β MSCs. (B) Bar graphs quantifying calcium in MSCs/TGF-β MSCs vs MSCs/TGF-β MSCs cultured in osteogenic medium. (C) Brightfield microscopy images of Oil Red O stained control and adipogenic differentiated MSCs and TGF-β MSCs. (D) Bar graphs quantifying Oil Red O absorbance in MSCs/TGF-β MSCs vs MSCs/TGF-β MSCs cultured in adipogenic medium. Error bars: mean ± SD. Unpaired, two tailed student's t test. Experiment was performed once with n=3 technical replicates. Scale bar in (A) = 500 µm; scale bar in (C) = 200 µm.

Supplemental Figure 2. TGF-β MSCs do not secrete TGF-β or sequester it on their cell surface after licensing. (A) MSCs were conditioned with TGF-β for 72 hours. The conditioned media was then removed and the cells were washed three times with PBS. Fresh media was placed on the cells and collected 24 hours and 48 hours later. Supernatants were assayed using a TGF-β-specific ELISA. (B) MSCs have been reported to retain latent TGF-β on their surface via the LAP/GARP complex. Untreated MSCs and TGF-β licensed MSCs were assayed for cell surface expression of both LAP and GARP using flow cytometry. Error bars: mean ± SD. Multiple unpaired, two tailed student's t test and One-way ANOVA, Tukey's Post Hoc test (n=3).

Supplemental Figure 3. Gene Ontology analysis of MSCs vs TGF-β MSCs. (A) Biological processes and (B) cellular compartment (Gene Ontology (GO) terms) circle graphic and enrichment score dot plots. The graphic shows the top 20 significantly differentially expressed genes paired with the top 9 GO terms after screening candidate genes (see materials and methods). The dot plot shows the enrichment score values of the top ten most significant enrichment terms.

Supplemental Figure 4. EP1 antagonism does not significantly affect TGF-β MSC ability to generate Tregs in MSC:T lymphocyte co-culture assays. TGF-β MSCs and TGF-β MSCs + EP1 Inhibitor (1 MSC to 10 lymphocytes) were co-cultured with anti-CD3/CD28 activated T lymphocytes for 96 hours. CD4+FoxP3+ Treg frequency was then assessed by flow cytometry. Error bars: mean ± SD. Multiple unpaired, two tailed student's t test and One-way ANOVA, Tukey's Post Hoc test (n=3).

Supplemental Figure 5. TGF-β MSCs can suppress pro-inflammatory activated macrophages *in vitro*. Untreated or TGF-β MSCs were co-cultured with activated bone marrow-derived macrophages for 72 hours. Macrophages had been previously activated by exposure to IFN-γ (100u/ml) for 24 hours followed by LPS (10ng/ml) stimulation for 4 hours. Stimulated macrophages cultured in the absence of MSCs served as the positive control. (A) MHC I, MHC II, CD80 and CD86 expression was analysed on macrophages by flow cytometry. Error bars: mean ± SD. *$p<0.05$, ***$p<0.001$. One-way ANOVA, Tukey's Post Hoc test (each dot represents an individual macrophage isolation, n=3). (B) Magnetic Luminex ® ELISA analysis of secreted levels of chemokines and pro-inflammatory cytokines in MSC/TGF-β MSC:macrophage co-culture assay supernatants. Secreted levels were assessed 96 h after co-culture. Error bars: mean ± SD. *$p<0.05$, **$p<0.01$ ***$p<0.001$, ****$p<0.0001$. One-way ANOVA, Tukey's Post Hoc test (each dot represents an individual macrophage isolation, n=3). (C) Individual cytokine-specific ELISAs were used to measure secreted levels of TNF-α and IL-10 from the same co-culture supernatants and at the same timepoint as detailed in part (B). Error bars: mean ± SD. **$p<0.01$, ***$p<0.001$. One-way ANOVA, Tukey's Post Hoc test (each dot represents an individual macrophage isolation, n=3).

Supplemental Tables

Item	Fluorochrome	Supplier	Cat. No.
CD3	FITC/PE	Biolegend, California, USA	100204/100205
CD4	APC/PE/Cy7	Biolegend, California, USA	100412/100527
CD8	APC	Biolegend, California, USA	100712
CD25	BV421	Biolegend, California, USA	102034
CD45.2	APC	Biolegend, California, USA	109814
CD62L	BV510	Biolegend, California, USA	104441
CD11b	FITC	Biolegend, California, USA	101205
CD11c	FITC	Biolegend, California, USA	117306
MHC I	PE/Cy7	Biolegend, California, USA	116621
Biotin MHC II	APC-Strep	Biolegend, California, USA	115003
CD80	BV421	Biolegend, California, USA	104725
CD86	BV510	Biolegend, California, USA	105039
PD-L1	APC	Biolegend, California, USA	124311
CD73	APC	Biolegend, California, USA	127210
CD105	APC	Biolegend, California, USA	120414
CD90.2	PE	Biolegend, California, USA	105308
Sca-1	APC	Biolegend, California, USA	108112
CD44	PE/Cy7	Biolegend, California, USA	103030
F4/80	PE	Biolegend, California, USA	123110
LAP	PE	Biolegend, California, USA	141306
GARP	eFluor 450	eBioscience/Thermo-Fisher Scientific, Dublin, Ireland	48-9891-82
Rat IgG1, κ Isotype Ctrl	APC	Biolegend, California, USA	400411
Rat IgG2a, κ Isotype Ctrl	APC	Biolegend, California, USA	400511
Mouse IgG2a, κ Isotype Ctrl	PE/Cy7	Biolegend, California, USA	400253
Rat IgG2b, κ Isotype Ctrl	PE/Cy7	Biolegend, California, USA	400617
Rat IgG2b, κ Isotype Ctrl	PE	Biolegend, California, USA	400607
Biotin Mouse IgG3, κ	APC-Strep	Biolegend, California,	401303

**Isotype Ctrl**		**USA**	
**Mouse IgG2a, κ Isotype Ctrl**	**APC**	**Biolegend, California, USA**	**400222**
**Rat IgG2a, κ Isotype Ctrl**	**PE**	**Biolegend, California, USA**	**400508**
**Armenian Hamster IgG Isotype Ctrl**	**FITC**	**Biolegend, California, USA**	**400906**
**Rat IgG2a, κ Isotype Ctrl**	**BV510**	**Biolegend, California, USA**	**400553**
**Armenian Hamster IgG Isotype Ctrl**	**BV421**	**Biolegend, California, USA**	**400935**
**Streptavidin**	**APC**	**Biolegend, California, USA**	**405207**
**SYTOX™ AADvanced™**	**PerCp**	**Thermo-Fisher Scientific, Dublin, Ireland**	**S10274**
**SYTOX™ Blue**	**BV421**	**Thermo-Fisher Scientific, Dublin, Ireland**	**S34857**

Table S1. Antibodies used for flow cytometry.

Item	Supplier	Cat. No.
**Recombinant Murine TGF-β1**	**Bio-Techne, Abingdon, UK**	**7666-MB-005**
**Recombinant Murine IFN-γ**	**Peprotech, London, Uk**	**315-05**
**LPS**	**Sigma Aldrich, Wicklow, Ireland**	**L2630**
**CellTrace Violet Cell Proliferation Kit**	**Thermo-Fisher Scientific, Dublin, Ireland**	**C34557**
**Dynabeads™ Mouse T-Activator CD3/CD28**	**Thermo-Fisher Scientific, Dublin, Ireland**	**11456D**
**SB431542**	**Cell Signalling Technology, MA, USA**	**14775S**
**Adenosine 5'-(α,β-methylene) diphosphate (AMP-CP)**	**Sigma-Aldrich, Wicklow, Ireland**	**M3763**
**SC-51322 (Selective EP1 antagonist)**	**Cayman Chemical, MI, USA**	**10010744**
**L-161,982 (Selective EP4 antagonist)**	**Cayman Chemical, MI, USA**	**10011565**
**Human/mouse TGF-β ELISA**	**eBioscience - Thermo-Fisher Scientific, Dublin, Ireland**	**88-8350**
**Mouse IL-10 ELISA**	**eBioscience - Thermo-Fisher Scientific, Dublin, Ireland**	**88-7314**
**Mouse TNF-α ELISA**	**eBioscience - Thermo-Fisher Scientific, Dublin, Ireland**	**88-7324**
**PGE2 ELISA**	**R&D Systems/Bio-Techne, Abingdon, UK**	**KGE004B**
**Mouse Magnetic Luminex Assay**	**R&D Systems/Bio-Techne, Abingdon, UK**	**LXSAMSM**

Table S2. Additional product information for specific reagents.

Reagent	Supplier	Product Code
**Alizarin Red S**	**Abcam**	**Ab146374**
**Ammonium Hydroxide**	**Sigma-Aldrich**	**338818**
**Ascorbic acid 2-P**	**Sigma-Aldrich**	**49752**
**Dexamethasone**	**Sigma-Aldrich**	**D4902**
**FBS**	**Hyclone**	**10309433**
**Hydrochloric acid**	**Sigma-Aldrich**	**H1758-100ML**
**Iscoves MEM**	**Sigma-Aldrich**	**13390**
**L-glutamine**	**ThermoFisher Scientific**	**25030024**
**L-thyroxine**	**Sigma-Aldrich**	**T2376**
**Methanol**	**Honeywell**	**34860-2.5L**
**Penicillin/streptomycin**	**ThermoFisher Scientific**	**15140122**
**StanBio Calcium Liquicolour Kit**	**ThermoFisher Scientific**	**SB-0150-250**
**B-glycerophosphate**	**Sigma-Aldrich**	**G9422**

Table S3. List of reagents used for MSC osteogenic differentiation induction and analysis.

Reagent	Supplier	Product Code
10% neutral buffered formalin	Sigma-Aldrich	HT501128
Dexamethasone 1mM	Sigma-Aldrich	D4902
DMEM (4.5g/L)	ThermoFisher Scientific	31966-021
FBS	ThermoFisher Scientific	10309433
Harris Haematoxylin	Sigma-Aldrich	HHS16-500ML
Indomethacin	Sigma-Aldrich	I7378
Insulin 1mg/ml	Sigma-Aldrich	I2643
Isopropanol	Sigma-Aldrich	I9516
MIX	Sigma-Aldrich	I5879
Oil Red O	Sigma-Aldrich	0-0625
Penicillin/streptomycin	ThermoFisher Scientific	15140122

Table S4. List of reagents used for MSC adipogenic differentiation induction and analysis.

Supplemental Methods

MSC differentiation assays

Osteogenic differentiation: Un-licensed or TGF-β-licensed Balb/c MSCs were seeded at a density of 20,000 cells per cm^2 in MSC medium (αMEM, 10% FBS, 1% penicillin/streptomycin) and cultured at 37°C in 5% CO2. Once confluent, medium was replaced with osteogenic induction medium. Control wells received normal MSC medium. Medium was replaced every 2 days, ensuring wells receive the correct medium. Observing the monolayer, cells were harvested before becoming detached. Osteogenic potential of the cells was determined based on intracellular calcium staining by Alizarin Red S. Medium was removed from wells and cells were washed with PBS. 95% methanol was used to fix the cells for 10 mins at room temperature. Cells were then washed with distilled water and 2% Alizarin Red S solution was added to the wells for 5 mins. Cells were then washed with distilled water and the monolayer was allowed to dry. A layer of water was added to the cells prior to imaging, which were acquired on an inverted light microscope. Cellular calcium content was quantified using the StanBIo Calcium Liquicolour kit. 0.5M HCL was added to wells and cells were harvested using a cell scraper and transferred to an Eppendorf. Cells/HCl solution was shaken overnight at 4°C. A working solution consisting of 1:1 binding reagent and working dye was prepared. Calcium standards ranging from 0-1ug/ml were made up, and 10ul of each standard or sample was added to 200ul working solution in a 96 well plate. Absorbance was measured at 595nm, and sample concentrations interpolated from the calcium standard curve. See Table S3 for the list of reagents used for MSC osteogenic differentiation induction and analysis.

Adipogenic differentiation: Un-licensed or TGF-β-licensed Balb/c MSCs were seeded at a density of 20,000 cells per cm^2 in MSC medium (αMEM, 10% FBS, 1% penicillin/streptomycin) and cultured at 37°C in 5% CO2. Once confluent, medium was replaced with adipogenic induction medium. Control wells received normal MSC medium. After 3 days, induction medium was removed and replaced with adipogenic maintenance medium for 1 day. The cells received 3 cycles of Adipogenic induction/maintenance medium, with the final maintenance medium left on cells for 5 days. After 5 days, medium was removed from wells and cells were washed with PBS and fixed with 10% neutral buffered formalin for 30 mins. Cells were then rinsed with distilled water and Oil Red O working solution was added to the cells for 5 minutes. To remove excess stain, cells were washed with 60% isopropanol, followed by distilled water. Hematoxylin was diluted 1:5 with distilled water and added to the wells for 1 minute. The cells were then rinsed with tap water, and covered with water for images to be taken on an inverted light microscope. Following imaging, Oil Red O was extracted by pipetting 99% isopropanol over the well surface several times. The isopropanol and dye was then transferred to an Eppendorf and centrifuged at 500 x g for 3 minutes to pellet debris. 200ul supernatant was placed per well of a 96 well plate and absorbance was measured at 490nm. See Table S4 for the list of reagents used for MSC adipogenic differentiation induction and analysis.
